# Exploring Intra- and Intermolecular Interactions in Selected *N*-Oxides—The Role of Hydrogen Bonds

**DOI:** 10.3390/molecules27030792

**Published:** 2022-01-25

**Authors:** Aneta Jezierska, Jarosław J. Panek, Kacper Błaziak, Kamil Raczyński, Aleksander Koll

**Affiliations:** 1 Faculty of Chemistry, University of Wrocław, ul. F. Joliot-Curie 14, 50-383 Wrocław, Poland; jaroslaw.panek@chem.uni.wroc.pl (J.J.P.); 298941@uwr.edu.pl (K.R.); 2 Faculty of Chemistry, University of Warsaw, ul. Pasteura 1, 01-224 Warsaw, Poland; kblaziak@chem.uw.edu.pl; 3 Biological and Chemical Research Center, University of Warsaw, Żwirki i Wigury 101, 01-224 Warsaw, Poland; 4 Non-Public Medical School in Wrocław, ul. Nowowiejska 69, 50-340 Wrocław, Poland; aleksander.koll@chem.uni.wroc.pl

**Keywords:** *N*-oxides, microsolvation, DFT, IEF-PCM, Fukui functions, SAPT, NBO, TD-DFT

## Abstract

Intra- and intermolecular interactions have been explored in selected *N*-oxide derivatives: 2-(N,N-dimethylamino-N-oxymethyl)-4,6-dimethylphenyl (**1**) and 5,5’-dibromo-3-diethylaminomethyl-2,2’-biphenol *N*-oxide (**2**). Both compounds possess intramolecular hydrogen bonding, which is classified as moderate in **1** and strong in **2**, and resonance-assisted in both cases. Density Functional Theory (DFT) in its classical formulation as well as Time-Dependent extension (TD-DFT) were employed to study proton transfer phenomena. The simulations were performed in the gas phase and with implicit and explicit solvation models. The obtained structures of the studied *N*-oxides were compared with experimental data available. The proton reaction path was investigated using scan with an optimization method, and water molecule reorientation in the monohydrate of **1** was found upon the proton scan progress. It was found that spontaneous proton transfer phenomenon cannot occur in the electronic ground state of the compound **1**. An opposite situation was noticed for the compound **2**. The changes of nucleophilicity and electrophilicity upon the bridged proton migration were analyzed on the basis of Fukui functions in the case of **1**. The interaction energy decomposition of dimers and microsolvation models was investigated using Symmetry-Adapted Perturbation Theory (SAPT). The simulations were performed in both phases to introduce polar environment influence on the interaction energies. The SAPT study showed rather minor role of induction in the formation of homodimers. However, it is worth noticing that the same induction term is responsible for the preference of water molecules’ interaction with *N*-oxide hydrogen bond acceptor atoms in the microsolvation study. The Natural Bond Orbital (NBO) analysis was performed for the complexes with water to investigate the charge flow upon the polar environment introduction. Finally, the TD-DFT was applied for isolated molecules as well as for microsolvation models showing that the presence of solvent affects excited states, especially when the *N*-oxide acceptor atom is microsolvated.

## 1. Introduction

Among the most important topics in the contemporary science is understanding of the role of intra- and intermolecular interactions and their influence on self-assembly of molecules and further on their diverse features observed at the macroscopic level [1,2,3,4,5,6]. Special attention has been paid to the non-covalent interactions because their nature still raises new questions; moreover, in the literature, one can find descriptions of their new kinds, e.g., [7,8,9,10]. The non-covalent interactions are ubiquitous in Nature [11,12,13]; therefore, they have become an object of many reviews, e.g., [14,15,16,17,18,19,20,21]. They significantly differ from covalent bonds because the electron sharing is not involved as a primary binding factor. They rather involve polarization or dispersive interactions between molecules or within a single molecule [22]. The non-covalent interactions are divided generally into electrostatic interactions, Van der Waals forces, π effects and hydrophobic effects [23]. Among electrostatic interactions, one can find ionic interactions, hydrogen and halogen bonds [24,25]. Let us focus on the hydrogen bonds, which could be divided into intra- and intermolecular. In addition, they could be classified as strong, moderate and weak [26,27,28]. They are crucial in biologically relevant systems as well as in new materials design, which are two important branches in the contemporary science [29]. The presence of intramolecular hydrogen bond is manifested by the structure stabilization as well as in the spectroscopic features, e.g., [30,31,32]. An interesting phenomenon associated with the hydrogen bond is proton transfer, which is significant in many processes at the molecular level, being responsible for structure changes and physico-chemical properties; see, e.g., [33,34,35,36,37,38,39]. The role of all intra- and intermolecular interactions has been intensively discussed because in many cases they are dynamic and their nature understanding is crucial in prediction and design of new compounds which could be novel drugs or materials. Moreover, to monitor some biologically important processes or self-assembly of molecules, it is necessary to enlarge our knowledge about them [40,41,42].

In the current study, we focused on intra- and intermolecular interactions in two *N*-oxides: 2-(N,N-dimethylamino-N-oxymethyl)-4,6-dimethylphenyl (**1**) [43] and 5,5’-dibromo-3-diethylaminomethyl-2,2’-biphenol *N*-oxide (**2**) [44]. *N*-oxides are interesting objects for experimental and theoretical studies because of their unusual chemical structure, possessing N→O bonds, which is involved in inter- or intramolecular hydrogen bond formation [45,46,47,48,49]. It was showed that the *N*-oxide group exhibits proton acceptor ability for hydroxyl, carboxyl, amine groups or water molecules. Moreover, it was reported on two types of *N*-oxides’ hydrogen bonding patterns:

(i) a single dimer and
(ii) a double acceptor bifurcated complex [50].

The NO bond in *N*-oxides, according to the general models, is classified as a donating type, denoted as N→O, in which the nitrogen atom shares its electron lone pair with the oxygen atom. The NO bond belongs to a class of strongly polar bonds because the charge separation exists between nitrogen (positive charge) and oxygen (negative charge) atoms. Recent studies of the *N*-oxide bond revealed that its nature is even more complicated [51,52,53]. The nitrogen atom provides its lone electron pair and forms a π-donating bond. The oxygen atom using its lone electron pairs is forming a π-back bond. Such back-bonding efficiency is strongly dependent on the substituents in the aromatic ring, e.g., pyridine ring substituents in para positions [50]. The situation described above holds usually for intermolecular hydrogen bonded complexes.

**Figure 1:**
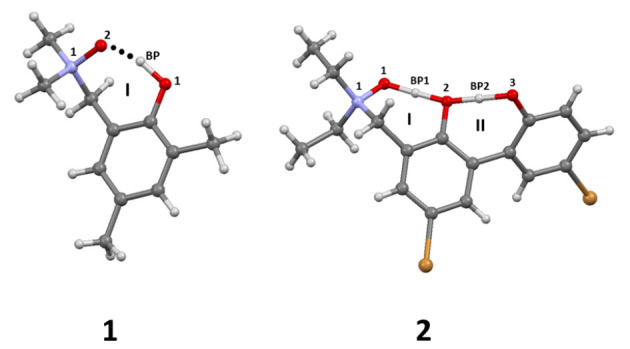
Molecular forms of studied *N*-oxides: 2-(N,N-dimethylamino-N-oxymethyl)-4,6-dimethylphenol (**1**) and 5,5’-dibromo-3-diethylaminomethyl-2,2’-biphenol *N*-oxide (**2**). The intramolecular hydrogen bridges are denoted O1-HBP…O2 in the case of **1** and O1-HBP1-O2 and O3-HBP2-O2 in the case of **2**. Atoms coloring and numbering scheme: nitrogen atom—blue, oxygen atoms—red, hydrogen atoms—white, carbon atoms—grey and bromine atoms—brown. BP—bridged proton. The presence of quasi-rings is denoted using I and II Roman numerals.

Here, we have analyzed two *N*-oxides with intramolecular hydrogen bond. Their molecular structures are presented in Figure 1. The first *N*-oxide, 2-(N,N-dimethylamino-N-oxymethyl)-4,6-dimethylphenol denoted as SEHBEM with database number: 593,412 in the Cambridge Crystallographic Data Center (CCDC) [54] possesses short intramolecular hydrogen bond with the O…O distance 2.541 Å [43]. The hydrogen bond is asymmetric with the bridged hydrogen atom located at the donor oxygen atom. In the crystal unit, a monohydrate form is present. The fact that the molecule crystallized as a monohydrate created a possibility to discuss the intermolecular interactions caused by the presence of water attached to the N→O group. The intermolecular hydrogen O…O interatomic distance is equal 2.718 Å. The symmetry and stability of the intramolecular hydrogen bond are affected by the presence of the water molecule at the proton acceptor side. The intramolecular hydrogen bond is stable in the water environment. Unfortunately, for the compound (**1**), it was impossible to obtain an anhydrous form [43]. However, the hydrogen bond present in 2-(N,N-dimethylamino-N-oxymethyl)-4,6-dimethylphenol (**1**) is weaker than that present in 2-(N-diethylamino-N-oxymethyl)-4,6-dichlorophenol [45,55]. Both compounds belong to the *N*-oxide Mannich base family. The X-ray diffraction measurements for the latter compound indicated the presence of two nonequivalent molecules in the asymmetric part of the unit cell with slightly different hydrogen bonds, with O…O interatomic distances of 2.407 Å and 2.426 Å, respectively [55]. The neutron diffraction redetermined the proton position in the hydrogen bridge and the O…O bridge lengths were reported to be equal 2.400 Å and 2.423 Å, respectively [56]. The weakening of the intramolecular hydrogen bond in (**1**) is not due to the water molecule presence, but the OH group acidity seems to be the most important factor [43]. The IR study of monohydrate showed a broad absorption above 1700 cm${}^{-1}$. There is a doublet at 3190 cm${}^{-1}$ and 3340 cm${}^{-1}$, which is present due to the water molecule. It was observed that, after the deuteration, the bands are shifted to 2500 cm${}^{-1}$ and 2380 cm${}^{-1}$. The broad band from 2000 cm${}^{-1}$ to 3100 cm${}^{-1}$ with a maximum at ca. 2300 cm${}^{-1}$ is associated with the presence of the O-H…O intramolecular hydrogen bond. After deuteration, the band is shifted to 1750 cm${}^{-1}$. It was reported that no absorption associated with strong hydrogen bond presence was observed in the low frequency region. An intense overtone band of $\gamma_{OH}$ at 1800 cm${}^{-1}$ should be detected. However, the $\sigma_{OH}$ band was found at 1270 cm${}^{-1}$, which was shifted after deuteration to 1010 cm${}^{-1}$. It was observed that, after the dehydration, the detected earlier doublet vanished. The $\nu_{OH}$ bond associated with the intramolecular O-H…O bridge shifted to lower frequencies, and it was followed by an intensification of the $\gamma_{OH}$ overtone band at 1800 cm${}^{-1}$ [43].

The second studied compound denoted as WUKMOE with the CCDC database number: 178615, possesses two intramolecular hydrogen bonds with different strength as reported by Wojciechowski et al. [44,57]. The crystal X-ray data indicated a presence of two strong intramolecular hydrogen bonds. The NO…H${}^{+}$…O${}^{-}$ hydrogen bond present between the *N*-oxide and O-H groups is very strong (the interatomic distance between the O…O atoms is 2.419 Å). It is most probably a barrierless intramolecular hydrogen bond with very flat and broad proton potential. The second intramolecular hydrogen bond formed between O-H groups was found asymmetrical as it was detected by FT-IR measurements. The molecular conformation of the molecule is non-planar due to a strong overcrowding effect derived from the presence of oxygen atoms involved in the intramolecular hydrogen bonds formation [44]. The FT-IR experimental measurements in solution and in the solid state are quantitatively comparable [44,57]. In both solvents, Wojciechowski et al. [57] reported a broad intense absorption in the region 800–1600 cm${}^{-1}$. The authors found that, in an acetonitrile solvent, the hydrogen bonds are slightly weaker. The finding is supported by NMR data, and it is characteristic for strong hydrogen bonds with broad flat proton potential with very small or non-existent barriers [58,59]. In the chloroform solution, there is a continuous absorption in the region 2550 cm${}^{-1}$–3300 cm${}^{-1}$. This absorption region is assigned to the OH…O${}^{-}⇌$ O${}^{-}$…HO intramolecular hydrogen bond, and, moreover, it was found to be characteristic for moderate hydrogen bonds with large proton polarizability (known in the literature as Zundel’s polarizability [60]). The main difference between IR data reported for measurements in solution and in the solid state is a continuum absorption at 2000 cm${}^{-1}$ associated with (most probably) a fast movement of the proton in the hydrogen bridges. It was found that the proton associated with the strong intramolecular hydrogen bond is more localized in the solid state than in the solution. A broad intense absorption at 800–1800 cm${}^{-1}$ region with a maximum at ca. 1000 cm${}^{-1}$ and a broadened band at 2400 cm${}^{-1}$ were observed. The spectral results showed that the favorable structure is proton-transferred form—from the O-H group to the *N*-oxide group. The O${}^{-}$…H${}^{+}$ON hydrogen bond is almost symmetrical, whereas the second O-H…O${}^{-}$ hydrogen bond is strongly asymmetrical [44].

We have investigated the two *N*-oxide derivatives using Car-Parrinello molecular dynamics (CPMD) [61] and Electron Localization Function (ELF) theory [62]. The CPMD simulations were performed in vacuo and in the crystalline phase. On the basis of the method, the intramolecular hydrogen bonds as well as spectroscopic features were studied. The CPMD results showed that the water molecule present in the crystalline form of the compound **1** is able to reattach at diverse acceptor sites (*N*-oxide and hydroxyl oxygen atoms). It is an interesting finding derived from the dynamical nature of the *N*-oxide-water complex. In the case of the compound **2**, the CPMD results revealed that the NO…H${}^{+}$…O${}^{-}$ hydrogen bond underwent frequent proton sharing and exchange events in vacuo. The crystalline phase simulation results showed the proton transfer to the acceptor side. In addition, the application of the ELF theory enabled us to investigate the electron density distribution along the hydrogen bond in both studied *N*-oxides [63]. It is worth underlining that our time-evolution findings have been in agreement with the experimental data described above [43,44,57].

Therefore, the main idea of the study is devoted to the detailed analysis of the N→O and intramolecular hydrogen bonds nature present in the *N*-oxides on the basis of Density Functional Theory (DFT) [64,65]. However, we have developed models for the electronic ground and excited states for monomers of the studied *N*-oxides. In the next step, the energy decomposition in the dimers and microsolvation models was carried out based on the Symmetry-Adapted Perturbation Theory (SAPT) method [66]. Finally, we have investigated the polar environment influence on the molecular features using microsolvation models.

## 2. Computational Methodology

### 2.1. Density Functional Theory (DFT) in the Electronic Ground State

**Figure 2:**
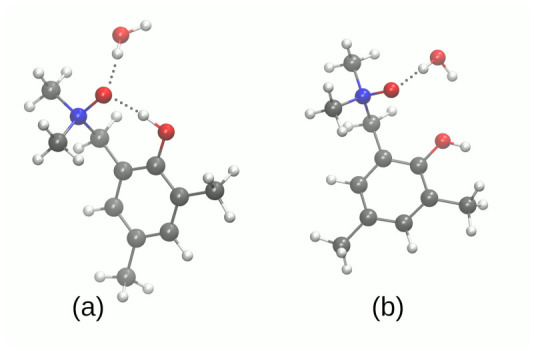
Conformers of the compound **1** (monohydrate form) computed at the B3LYP/6-311++G(d,p) level of theory. (**a**) closed form; (**b**) open form.

**Figure 3:**
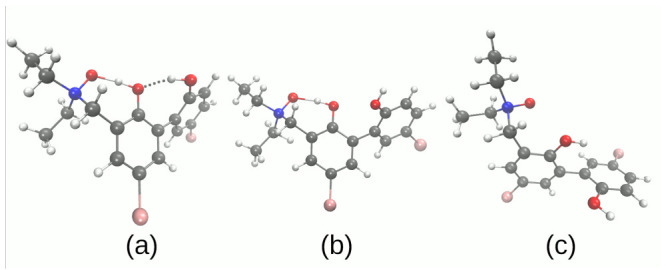
Conformers of the compound **2** computed at the B3LYP/6-311++G(d,p) level of theory. (**a**) closed form; (**b**) with one hydrogen bond; (**c**) open form.

The molecular forms of the investigated *N*-oxides: 2-(N,N-dimethylamino-N-oxymethyl)-4,6-dimethylphenyl (**1**) and 5,5’-dibromo-3-diethylaminomethyl-2,2’-biphenol *N*-oxide (**2**) are showed in Figure 1 and Figure S1. The models of the studied compounds were constructed on the basis of their crystal structures [43,44]. The presence of the intramolecular hydrogen bonds resulted in the quasi-rings formation, which is denoted in Figure 1 by I and II Roman numerals. The quantum-chemical simulations were performed in a framework of Density Functional Theory (DFT) [64,65] in vacuo and with solvent reaction field (Polarizable Continuum Model – IEF-PCM formulation and water as a solvent) [67,68]. In the case of the *N*-oxide **1**, the models with water molecule (as indicated in the crystal structure) as well as single molecule were built and studied theoretically. The geometry minimization was carried out using B3LYP [69], CAM-B3LYP [70], M05-2X [71] and ωB97XD [72] functionals to reproduce the metric, energetic and electronic structure parameters. The 6-311++G(d,p) triple-zeta split valence basis set by Pople et al. [73,74] was applied for this purpose. The harmonic frequencies were calculated to confirm that the obtained structures correspond with minima on the Potential Energy Surface (PES). We have investigated so called closed (with intramolecular hydrogen bond) as well as open forms of the *N*-oxides (see Figure 2 and Figure 3). Next, the reaction path of the bridged hydrogen was scanned (with 0.05 Å increment of the OH-ON distance) and with the valence angle frozen (which defines the intramolecular hydrogen bond) while the remaining parts of the molecules were optimized. On the basis of the simulations, we have obtained a set of energies, which were further used to construct potential energy profiles. This part of the simulations was performed using the Gaussian 09 Rev. D.01 suite of programs [75]. The atomic nucleophilicity *f*${}^{-}$(*r*) and electrophilicity *f*${}^{+}$(*r*) indices were calculated at each point of the proton transfer reaction pathway between the Min_1 and Min_2 structures (O-H bond distance elongation). The electron density population was computed using a Hirshfeld method [76] implemented in the Gaussian 16, Rev. A.03 package [77] using the CAM-B3LYP/6-311++G(d,p) level of theory [70,73,74] for further application in Fukui functions computations [78]. The local atomic indices of nucleophilicity and electrophilicity were calculated using Fukui functions described as:

(1) $$f^{-}\left(r\right)=\rho_{N}\left(r\right)-\rho_{\left(N-1\right)}\left(r\right)$$

(2) $$f^{+}\left(r\right)=\rho_{\left(N+1\right)}\left(r\right)-\rho_{N}\left(r\right)$$

where $\rho_{N}$(*r*), $\rho_{\left(N-1\right)}$(*r*) and $\rho_{\left(N+1\right)}$(*r*) are electron densities, respectively, for *N* electrons, *N*-1 electrons and *N*+1 electrons species. The Fukui functions, however, were computed only for the compound **1** (single molecule and monohydrate complex) because the chemical composition of the compound **2** made us unable to obtain reliable results.

**Figure 4:**
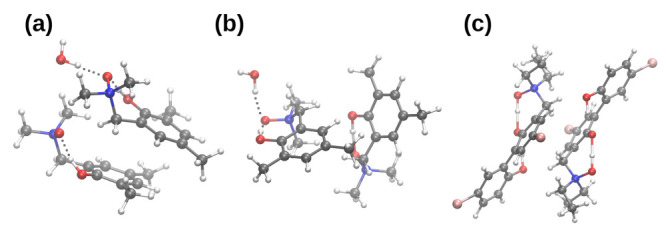
Dimers investigated in the SAPT study. (**a**) dimer 1 of the compound **1** with water molecule; (**b**) dimer 2 of the compound **1** with water molecule; (**c**) dimer 1 of the compound **2**.

**Figure 5:**
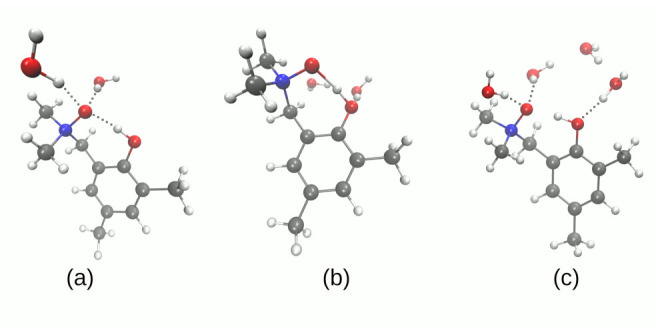
Microsolvation models of the compound **1**. (**a**) two water molecules on the acceptor side, **1**-A2; (**b**) one water molecule on the acceptor side and one on the donor side, **1**-AD; (**c**) four water molecules, two on the acceptor and two on the donor sides, respectively, **1**-A2D2.

**Figure 6:**
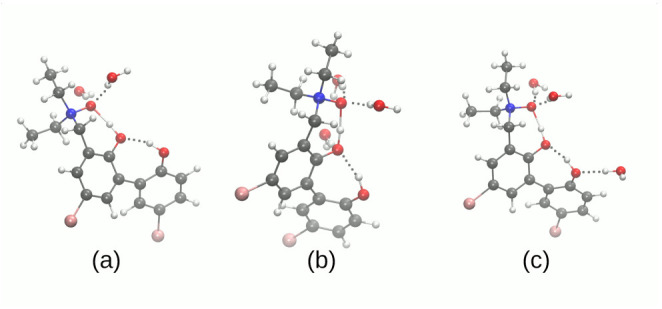
Microsolvation models of the compound **2**. (**a**) two water molecules on the O1 (acceptor atom) side, **2**-A2; (**b**) two water molecules on the O1 acceptor side and one water molecule on the O2 donor side, **2**-A2D${}_{A}$; (**c**) two water molecules on the O1 acceptor side and one water molecules on the O3 donor side, **2**-A2D${}_{B}$.

Next, the models of dimers of the compounds **1** and **2** (see Figure 4) were constructed based on the X-ray data as described above [43,44]. The geometry optimization was performed at the ωB97XD/6-311++G(d,p) level of theory [72,73,74]. Finally, the microsolvation models were built with 1–4 water molecules located at the donor or acceptor sides (for details, see Figure 5 and Figure 6 as well as Figures S3 and S4). The energy minimization for the complexes was performed using B3LYP and ωB97XD functionals and 6-311++G(d,p) basis set [69,72,73,74]. The harmonic frequencies were computed as well and no imaginary frequencies were detected. Furthermore, the dimers and microsolvation models were used for Natural Bond Orbitals (NBO) (with special emphasis put on the atomic charge flow) [79], Symmetry-Adapted Perturbation Theory (SAPT) [66], and Time-Dependent Density Functional Theory (TD-DFT) [80] investigations. This part of the computations was performed with the Gaussian 16 Rev. A.03 suite of programs [77]. The graphical presentation of the results was prepared with the assistance of the VMD 1.9.3 [81] program.

### 2.2. Symmetry-Adapted Perturbation Theory (SAPT) Protocol for Energy Decomposition in Dimers and Clusters

Decomposition of the interaction energy in the dimers and microsolvated structures has been carried out on the basis of Symmetry-Adapted Perturbation Theory (SAPT) [66]. This scheme is a perturbative calculus, differing from the general Møller-Plesset (MP) theory in many aspects, the most important being the splitting of the Hamiltonian into diverse parts. While the MP scheme uses the following choice (in simplified terms):

(3) $$\hat{H}={\hat{H}}_{0}+\hat{V},$$

where ${\hat{H}}_{0}$ is the unperturbed so-called “shifted Fock operator”, and $\hat{V}$ is the correlation operator, the SAPT scheme enforces the following partitioning:

(4) $$\hat{H}={\hat{F}}_{A}+{\hat{W}}_{A}+{\hat{F}}_{B}+{\hat{W}}_{B}+\hat{V},$$

where subscript indices A and B denote the operators corresponding to the two respective monomers, $\hat{F}$ and $\hat{W}$ are the Fock and intra-monomer correlation parts of the Hamiltonian, and $\hat{V}$ is the inter-monomer term. This allows for a strict control of the perturbation order within as well as between the monomers. The two-digit code determines that order, so that a given term E${}^{ij}$ corresponds to the *i*-th intermolecular and *j*-th intramolecular perturbation order. Furthermore, the energy terms are labelled according to their physical meaning (in general: electrostatic interaction of frozen electron densities of the monomers, Pauli exchange repulsion, mutual polarization–induction, and dispersion). The basis set superposition error (BSSE), which is associated with supramolecular calculations, is formally not present in the SAPT perturbative treatment when the calculations are carried out in a full dimer basis set—such approach was used in our study. The residual Hartree–Fock term includes the BSSE according to the Boys–Bernardi method [82]. The details of the SAPT scheme are given in an original review [66] as well as in papers describing recent implementations used in this work [83,84].

In the current study, the structures of dimers and microsolvated molecules of compounds **1** and **2** optimized at the ωB97XD/6-311++G(d,p) level of theory were used for the SAPT calculations. Additionally, the dimers in the X-ray experimental configurations were also included. The SAPT2 level of SAPT approximation, as implemented in the PSI4 ver. 1.3.2 code [85], was used in conjunction with jun-cc-pVDZ basis set (a truncation of aug-cc-pVDZ basis [86,87]).

### 2.3. Time-Dependent Density Functional Theory (TD-DFT)

Among the levels of theory applied within the current study, the CAM-B3LYP/6-311++G(d,p) level was selected to model excited state properties in the gas phase for the isolated and microsolvated molecules of compounds **1** and **2**. The CAM-B3LYP functional [70] was devised to overcome the problems of the B3LYP functional to describe the charge-transfer excited states within the time-dependent DFT (TD-DFT) approach [88]. The 25 singlet and 25 triplet states were included in the excitation space, and the model of vertical excitation (at the ground state equilibrium geometry) was assumed. The TD-DFT calculations were carried out with the Gaussian 16, Rev. A.03 package [77].

## 3. Results and Discussion

### 3.1. Structural, Energetic and Reactivity Properties of the N-Oxides

The first part of the results analysis is devoted to the structural, energetic and reactivity properties of isolated molecules of compounds **1** and **2** (see Figure 1), including monohydrate of **1**, as found in its crystal structure [43]. Detailed numerical data and depiction of the analyzed structures are provided in Figure 2, Figure 3 and Figure S1 and Tables S1–S4 of the Supplementary Material file, and this section contains only selected, most significant conclusions resulting from the analysis of these data.

The intramolecular hydrogen bond of crystalline **1** is accompanied by the intermolecular contact to the water molecule present in the crystal structure. The DFT study for isolated **1** as well as its monohydrate (Tables S1 and S2, respectively) shows, however, a common trend. The intramolecular O1…O2 bridge length is well reproduced to within ±0.05Å in both gas phase and the PCM solvent model, but the polarizing effect of the PCM results in the bridge shortening by ca. 0.02 Å. This bridge length decrease is well conserved among the four tested functionals, and is also kept in the presence of an explicit water molecule. Even more interesting is the fact that the intermolecular O2…O bond is shortened more strongly, by ca. 0.04–0.05 Å. In all cases, however, the proton remains at the donor side. A quite different situation is recorded for the compound **2**, which experiences small O1…O2 bridge lengthening (by less than 0.01 Å) under the inclusion of the PCM solvation. The second bridge of the compound **2** behaves again as noted for **1**, being shortened by 0.02 Å. Unusual behavior of the O1…O2 hydrogen bond is related to the fact that the PCM solvation enforces proton transfer in this bridge, underlining its strength and flatness of the proton potential function [63]. This is supported by the energetic data from Table S4. The energy difference between the “closed” and “open” forms is not a rigorous energy of an intramolecular hydrogen bond, but rather its estimate only; however, it is a useful approximation especially for a series of related structures treated with a uniform protocol, as is the case in this study. On the basis of Table S4, we can estimate that the energy of intramolecular hydrogen bond in isolated **1** is ca. 13 kcal/mol, and the presence of the PCM solvent decreases this energy to ca. 10 kcal/mol. It might seem paradoxical that the weakened bond is shorter, but this can be explained: the “open” form enables interaction of the free, non-bonded hydroxyl group with the dielectric medium. The stability of the “open” form is thus increased, and the intramolecular hydrogen bridge appears less stable. Can this reasoning be also applied to the compound **2**? Indeed yes; the energy difference between conformers (a) and (b) (see Figure 3) corresponds to the estimate of the O2…O3 bridge strength, and it is ca. 6 kcal/mol in the gas phase, but 5 kcal/mol in the PCM solvent field. This is a marginal effect, but the difference between conformers (b) and (c), corresponding to the stronger O1…O2 bridge, is ca. 15 kcal/mol in the gas phase and 11 kcal/mol when the PCM is applied. Such results prompted us to investigate details of the proton potential profiles in the hydrogen bridges, as well as examine closely the electronic structure parameters for the intramolecular hydrogen bond using the tools of conceptual DFT, namely Fukui functions.

**Figure 7:**
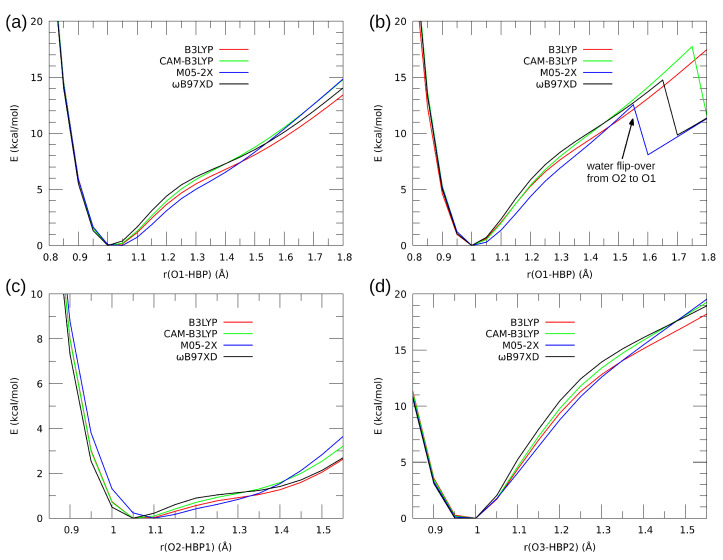
Proton potential profiles for the hydrogen bonds in the investigated systems—results of DFT relaxed scans. (**a**) compound **1** without water molecule; (**b**) compound **1** as monohydrate; (**c**) compound **2**—bridge O1…O2; (**d**) compound **2**—bridge O2…O3.

The proton potential profiles for the investigated compounds, shown in Figure 7, indicate that, in case of the compound **1** (its single molecule as well as the monohydrate), the proton transfer to the acceptor side is not favored, and a single minimum at the donor atom with only an inflexion point close to the acceptor atom are found. However, the role of single water molecule is very significant, as shown in Figure 7b: the relaxed potential energy scans, including full optimization of the non-scanned parameters, allowed the water molecule to change the acceptor atom it is attached to. Initially, this acceptor is O2 of the *N*-oxide moiety, but at some point (when the HBP proton is already on the O2 side) the water molecule undergoes reorientation, flips over the molecule of **1**, and forms a hydrogen bond to the O1 hydroxyl atom. This fact is in total agreement with our CPMD study where similar reorientation was registered in a dynamical context [63]. Furthermore, also one of the two hydrogen bonds in the compound **2**, namely the O3-HBP2…O2 bridge, does not favor the proton transfer. The role of this bridge is strengthening of the other, O2-HBP1…O1, bridge which possesses a very flat potential profile. The DFT scans indicate the existence of large delocalization of the HBP1 proton, which is expected even in the gas phase. A final remark before proceeding to the Fukui function study is the performance of diverse DFT functionals. Figure 7 shows clearly that the differences between the profiles are very minor, and the only issue which could be significant is the point of the water molecule flip-over, not registered by B3LYP and recognized most early by M05-2X functional.

**Figure 8:**
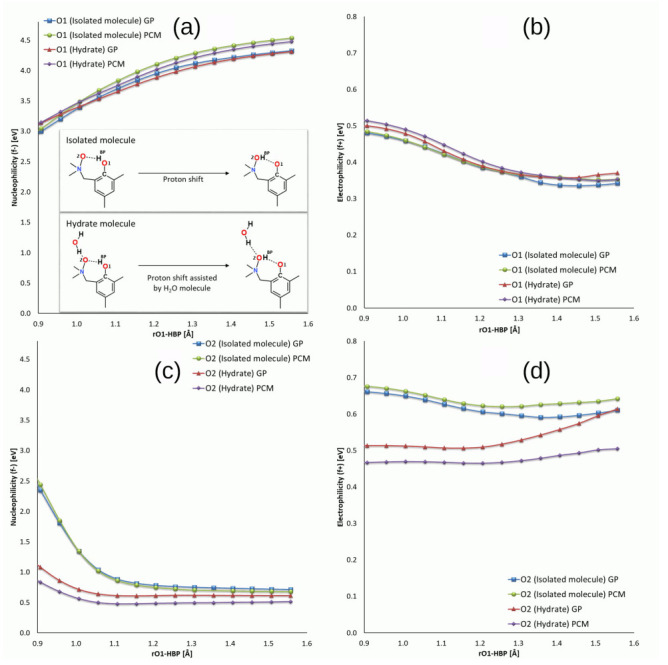
Calculated Fukui functions describing changes of nucleophilicity of O1 (top left, (**a**)) and O2 (bottom left; (**c**)) oxygen atoms and electrophilicity of O1 (top right, (**b**)) and O2 (bottom right, (**d**)) oxygen atoms along the proton HBP shift pathway. HBP—bridged proton, GP—gas phase, PCM—simulations with Polarizable Continuum Model and water as a solvent.

The Fukui functions [89] have been applied to the atomic systems (**1** in Figure 1) in order to follow the electrophilicity (*f*${}^{+}$) and nucleophilicity (*f*${}^{-}$) changes related to acceptor (O2) and donor (O1) oxygen atoms during the proton (HBP) movement. The CAM-B3LYP/6-311++G(d,p) level of theory was used to investigate two analogous structures: isolated molecule and its hydrate which contains one extra water molecule associated with the acceptor oxygen atom. Both atomic systems were investigated by using two theoretical approaches: in the gas phase (GP) and surrounded by implicit solvent dielectric potential (PCM, dielectric potential for water). The reactivity indices’ changes were studies as a function of HBP-O1 bond distance. As it is shown in Figure 8a, the significant increase in the nucleophilic character of the proton donor has been observed while the proton was distancing from the O1 oxygen. The nucleophilicity has increased from 3.00 eV to 4.33 eV for the isolated molecule in the GP, from 3.04 to 4.54 eV for the molecule under the PCM approximation, from 3.13 to 4.32 eV for the monohydrate in the GP, and from 3.15 to 4.48 eV for monohydrate with the assistance of the PCM. No significant impact of an extra water molecule (in monohydrate complex) on this factor has been noticed; however, a subtle difference has been observed between the gas phase and PCM approximations. The electrophilicity calculated for the donor oxygen atom O1 decreased smoothly along the reaction path similarly for all types of the investigated systems from 0.48–0.51 eV to 0.34–0.37 eV (Figure 8). The presence of an extra water molecule coordinated through the hydrogen bond to the atom O2 (Figure 8c,d caused the changes of the proton acceptor (oxygen atom O2) orbitals which could be observed by different reactivity behavior. In the case of the nucleophilicity changes of the acceptor proton O2, for all the molecular systems, a smooth decrease of the potential has been observed. The proton acceptor O2 in the isolated molecules, computed both in the GP and PCM, possesses much higher nucleophilic character than their monohydrated counterparts. The nucleophilicity of the O2 atom for the systems has decreased while the proton approaches from 2.44 and 2.35 eV to 0.68 and 0.71 eV. The extra water molecule attached to the acceptor oxygen atom significantly decreased the initial nucleophilic potential deposited on the O2 atoms in both GP and PCM approximations. The *f*${}^{-}$ potential computed for the monohydrate atomic systems decreased smoothly from 1.08 to 0.61 eV and 0.84 to 0.52 eV for GP and PCM, respectively. This phenomenon is caused by the presence of an extra water molecule deposited in the neighbourhood of the proton acceptor O2 atom. The water molecule interacts with the acceptor O2 atom from the other site of the intramolecular hydrogen bridge (introducing the competition in the interactions) and compensates its nucleophilic potential. For the isolated molecules, the electrophilic potential computed for the single acceptor oxygen O2 atom slightly decreased for both GP and PCM models from 0.66 to 0.61 eV and from 0.68 to 0.64 eV, respectively (Figure 8d). The contrary behavior has been observed for the O2 atom located in the monohydrated form of the compound **1** computed in both GP and PCM formalisms. The electrophilic character of the proton acceptor (O2) visibly increased during the proton (HBP) movement within the intramolecular hydrogen bridge from 0.47 to 0.51 eV for the PCM and from 0.51 eV to 0.61 eV in the gas phase. In this case, the explicit presence of the extra water molecule also plays a key role in elevating an electrophilic potential of selected acceptor oxygen atom during the bridged proton (HBP) movement. The closeness of the second proton belonging to the water molecule provided the local electron deficiency around the O2 oxygen atom making it more prone to accept the electrons (increase in electrophilicity).

### 3.2. Self-Assembly and Microsolvation of the Studied N-Oxides

Molecular self-assembly, a process governed by non-covalent interactions, leads ultimately to condensed phases (liquid, amorphous solid, crystal). These processes, however, start at the most basic level of dimerization, and this is the point of view we take in this part of the study. Three structures of dimers are considered (see Figure 4). Two structures for **1** differ by the relative position of monomers. In the dimer 1 of **1**, the molecules are arranged in an anti-parallel mode, while in the dimer 2, the molecules are skewed. The dimers, derived from the crystal structure, are not bound by intermolecular hydrogen bonds—the presence of relatively strong intramolecular hydrogen bridges seems to screen the donor and acceptor atoms and prevent their interaction with the adjacent molecules. However, in case of **1**, a monohydrate is formed in the solid state, and we will analyze the impact of the presence of the water molecule on the properties of the dimers.

At the beginning, we would like to underline that, because of the size of the molecules and the presence of heavy atoms, it was possible to apply the SAPT decomposition to the dimer of **2** only at the SAPT0 level, which does not include intramonomer electron correlation. Our experience, supported by the data for **1** presented in Table 1, indicates that, in most cases, SAPT0 predicts the intermolecular bonding which is stronger than at the SAPT2 level. The comparison of SAPT0 and SAPT2 results for **1** suggests that SAPT2 interaction energy for the dimer of **2** would be close to –30 kcal/mol, amounting to the interaction ca. five times more strong than for **1**.

The comparison of the interaction energy components for the dimers taken directly from the X-ray structures [43,44] and those from the DFT optimization show that the dimer with the experimental structure is less strongly bound. The differences between the obtained structures are shown in Figure S2 of the Supplementary Material. Generally, the spatial orientation has been preserved, but the molecules shifted during the optimization procedure to maximize the direct contact. This result is not unexpected because the presence of other molecules in each spatial direction forces a given molecule to divide its interaction capabilities among more than only one neighbour. The experimentally determined crystal structure prefers weak intermolecular forces, i.e., dispersion, to provide the most important contribution to the molecular binding. It is also important to note that **1** is packed in the crystal in such manner that induction is not significant. The comparison of the dimers of **1** and **2** based on the X-ray data requires a careful analysis of the ratios between the interaction energy components. For example, while for **1** the dispersion is ca. two times larger than electrostatics, for **2**, it is not larger but smaller. These relations are not conserved after the DFT optimization; we consistently observe that electrostatics and dispersion terms are almost equal. An induction plays a rather minor role, but the exchange (Pauli repulsion) grows strongly, signifying that the molecules are closer to each other than in the experiment. The presence of the hydrate (additional water molecule) in the crystal of **1** makes virtually no impact on the structure of dimer type 2 because the water molecule in this dimer type is screened from direct interaction with the other monomer. In case of the dimer type 1, the water molecule brings the same amount of (attractive) electrostatics as (repulsive) exchange, so the effects of hydration water on these two terms cancel out. The strengthening of the interaction by 2.6 kcal/mol results from increased induction and dispersion.

The computational studies of microsolvation provide unique opportunity to determine which centers of interaction are more affected by the presence of solvent (water) molecules, and reveal charge flow upon microsolvation and polarization by water molecules. The microsolvation models are shown in Figure 5 and Figure 6 as well as in the Supplementary Material—Figures S3 and S4. The metric parameters of the intra- and intermolecular hydrogen bonds obtained as a result of microsolvation are presented in Tables S5–S7, respectively. The simulations were performed using two functionals: hybrid B3LYP [69] and ωB97XD [72]. The second functional has implemented dispersion; therefore, it was of interest to benchmark its performance in the microsolvation study. The metric parameters of the intra- and intermolecular hydrogen bonds provide an overview of the possible non-covalent interactions introduced by the water molecules in the vicinity of the proton-donor or proton-acceptor atoms. This could be useful in the design of new *N*-oxide type compounds with desired properties associated with the intramolecular hydrogen bridge modulation. Figure 5 and Figure 6 depict the systems used in the SAPT and NBO microsolvation studies, labeled according to the sites the water molecules interact with. Thus, label A2 signifies two water molecules hydrogen-bonded to the *N*-oxide oxygen atom (acceptor of intramolecular O-H…O hydrogen bond); label AD signifies two water molecules, one hydrogen-bonded to the *N*-oxide oxygen atom, and another to the hydroxyl donor atom; A2D2—microsolvation by four water molecules, two at the acceptor site and two at the donor site. Additionally, for the compound **2**, the designations D_A_ and D_B_ denote which hydroxyl group is affected by the water molecule: respectively, the donor to the *N*-oxide moiety or the O3 atom.

The SAPT analysis of the microsolvation models (the results presented in the bottom part of Table 1) shows that the interaction of the water molecules with **1** and **2** is most effective when the acceptor, rather than donor, atom is hydrated. The comparison of the components of the total interaction energy shows that the induction term is responsible for this behavior. This is best visible in the case of **1**-A2 (a molecule of **1** and two water molecules at the acceptor site) as compared to **1**-AD (a molecule of **1**, one water molecule at the acceptor site and another at the donor site). The change in electrostatic term is compensated by the exchange repulsion, and the dispersion forces change only slightly, but the induction is more beneficial for the **1**-A2 case (−12.66 kcal/mol, vs. −8.50 kcal/mol for **1**-AD). The same can be stated about the **2**-A2 and **2**-A2D systems, where addition of a water molecule at the donor site does not bring significant strengthening of the microsolvated complex. The notion of “acceptor site is more susceptible to external interactions” should be taken into account during the rational design of *N*-oxide based structures. The SAPT study shows the interaction energies, which are molecular properties and cannot be directly analyzed in terms of specific atoms. For this reason, we have carried out electronic structure discussion using the NBO approach.

Table 2 presents changes in the NBO atomic charges of the atoms involved in the hydrogen bonding network upon interaction with water molecules. Isolated molecules of the compounds **1** and **2** are the baseline systems for comparison purposes. The first notion to be observed is that the N1 atom of the *N*-oxide moiety is almost unaffected by microsolvation; moreover, it is almost neutral. Relatively large changes of the NBO charge on the acceptor oxygen atom (up to 0.05 e) do not affect the N1 atom. Even more surprising is that the same can be said about the bridge hydrogen atoms—HBP1 (and HBP2 for **2**)—their net NBO charges do not change more than 0.01 e in diverse microsolvation schemes. These changes, moreover, do not follow common patterns; for example, the A2 microsolvation (two water molecules interacting with the *N*-oxide acceptor oxygen atom) results in a small decrease in the HBP1 atomic charge for **1**-A2, but a small increase in the case of **2**-A2. It is thus more interesting to concentrate on the *N*-oxide acceptor and hydroxyl donor oxygen atoms. Table 2 shows that the presence of interacting water molecules increases the polarization of the relevant oxygen atom, making it more negative. Each of the oxygen atoms reacts more strongly upon direct hydrogen bonding interaction with a water molecule, but the charge flow is present also when e.g., the acceptor atom is considered, but the water molecules are added to the donor moiety—see, especially, the *N*-oxide oxygen atom in the **1**-A2 vs. **1**-A2D2 systems, or in the **2**-A2 vs. **2**-A2D${}_{A}$ complexes. The last two cases show also one more feature of the microsolvation—the interaction saturation effect. The *N*-oxide acceptor oxygen atom is most perturbed when it is the sole target for water molecules (scenarios **1**-A2 and **2**-A2), but when the donor oxygen atom is also hydrogen-bonded to water molecules, the *N*-oxide acceptor loses some electron density.

### 3.3. Impact of Microsolvation on Electron Excitations in the Studied *N*-Oxides

The presence of solvent can affect excited states of a given system in a number of ways. First, interactions (electrostatic, hydrogen-bonded and other non-covalent directional bonds, van der Waals) between the solute and solvent can affect the orbital levels of the solute, modifying accordingly the excitation energies. This effect grows stronger when the interaction with solvent is stronger as well. Second, the solvent can be a target or source of excitation (charge transfer to the solvent molecule), thus effectively changing the excitation mechanism. The results gathered below in Table 3 point rather to the first mechanism because the excitation energies are not dramatically affected, although systematic shifts are observed.

The absorption energies of the first three singlet excited states are shifted upwards, resulting in a decrease in the absorption maximal wavelength. These hypsochromic shifts are not large—for the vertical absorption leading to the S${}_{1}$ state, up to 10 nm for microsolvated **1** and 7 nm for the microsolvated **2**. They are, however, quite systematic. The case of **1** shows that the microsolvation of both the donor and acceptor atoms is important: when two water molecules are coordinated to the O2 acceptor atom, the absorption wavelength falls down from 261.12 nm to 257 nm, but when one water molecule is attached to the O1 donor, and another water molecule coordinates the O2 atom, the hypsochromic effect is even larger (the S${}_{1}$ excitation is at 254 nm). The full saturation of the hydrogen bridge solvation shell (two water molecules at O1 and two more at O2) brings the wavelength down to 251.5 nm. These results for **1** are consistent with the data for **2**, where the saturation of the O1 acceptor atom with two water molecules brings the absorption wavelength from the gas-phase value of 276.6 nm down to 272 nm, and addition of a third water molecule to the O2 donor yields further hypsochromic shift to 269.1 nm. In all cases, the oscillator strength is also decreased upon microsolvation, and this quenching is more pronounced for **1**—by almost one third, while for **2** by ca. 15%. Summarizing the excited state microsolvation study, one should note that the impact of water molecules is somewhat different from the SAPT investigation (which preferred interactions between water and the *N*-oxide acceptor atom): the hypsochromic shift is larger for the **1**-AD than for **1**-A2.

## 4. Conclusions

The metric parameters and energetic analysis of intra- and intermolecular hydrogen bonds revealed that spontaneous proton transfer phenomena do not occur in the electronic ground state for the isolated molecule (**1**) as well as its monohydrated complex. In the case of the compound **2**, a proton transfer phenomenon was observed confirming the flatness of the PES reported in the literature. The PES profile possesses two energy minima and the proton transfer process is favored especially in the N1-O1…HBP1-O2 hydrogen bridge. The proton position in the intramolecular hydrogen bond of **1** is coupled with the orientation of the water molecule in the monohydrate, and the proton transfer process in the bridge leads to the water molecule flip-over.

The electronic structure of the studied *N*-oxides was analyzed using Fukui functions describing electro- and nucleophilicity. Following the main motif of the study, the impact of hydrogen bonding on the molecular properties, it was found that the explicit presence of the water molecule (microsolvation) results is elevating an electrophilic potential of the acceptor oxygen atom during the proton (HBP) movement: the presence of water molecule provides local electron deficiency at the O2 oxygen atom making it more prone to accept the electrons (increasing its electrophilicity).

The interaction energy partitioning according to the SAPT scheme revealed a rather minor role of induction (mutual polarization) in the formation of homodimers. However, the same induction term is responsible for the preference of water molecules to interact with *N*-oxide hydrogen bond acceptor atoms in the microsolvation study.

The effects of microsolvation on the electronic excitations (UV/Vis spectra) are not large, but systematic: hypsochromic shifts of 10 nm are observed already with four water molecules in the solvation shell.

## Figures and Tables

**Table 1:** SAPT interaction energy partitioning at the SAPT2/jun-cc-pVDZ level of theory. All terms in kcal/mol.

System	Electrostatics	Exchange	Induction	Dispersion	SAPT0	SAPT2
Dimers based on X-ray coordinates, no water molecules						
**1**, dimer 1	−4.23	7.47	−1.65	−9.22	−8.28	−7.63
**1**, dimer 2	−3.71	3.91	−1.12	−4.99	−6.49	−5.91
**2**, dimer 1	−20.29	13.06	−6.62	−18.54	−32.40	-
DFT−optimized dimers, with or without one water molecule for **1**						
**1**, dimer 1+H${}_{2}$O	−24.27	35.21	−6.92	−22.87	−21.34	−18.86
**1**, dimer 1	−19.65	30.54	−5.61	−21.51	−18.34	−16.23
**1**, dimer 2+H${}_{2}$O	−13.60	19.25	−3.72	−13.62	−13.28	−11.69
**1**, dimer 2	−13.59	19.37	−3.70	−13.62	−13.16	−11.53
**2**, dimer 1	−26.93	30.55	−9.24	−28.79	−34.41	-
Microsolvation						
**1**-A2	−39.21	41.75	−12.66	−8.34	−22.70	−18.46
**1**-AD	−27.14	30.41	−8.50	−7.87	−16.74	−13.10
**1**-A2D2	−52.45	54.36	−16.56	−12.52	−33.36	−27.17
**2**-A2	−35.39	39.12	−11.22	−8.71	−20.34	−16.21
**2**-A2D${}_{A}$	−41.02	45.96	−12.47	−12.58	−24.99	−20.11
**2**−A2D${}_{B}$	−45.47	50.41	−14.26	−11.30	−26.11	−20.62

**Table 2:** NBO atomic charges for the atoms involved in hydrogen bonding network, calculated at the ωB97XD/6-311++G(d,p) level of theory for the microsolvation models.

System	*N*-Oxide Moiety		Donor –OH 1		Donor –OH 2	
	N1	O2 in 1, O1 in 2	HBP1	O1 in 1, O2 in 2	HBP2	O3
**1**	−0.036	−0.737	0.507	−0.722	-	-
**1**-A2	−0.032	−0.767	0.505	−0.722	-	-
**1**-AD	−0.033	−0.749	0.516	−0.764	-	-
**1**-A2D2	−0.030	−0.759	0.514	−0.788	-	-
**2**	−0.042	−0.713	0.508	−0.754	0.503	−0.711
**2**-A2	−0.040	−0.765	0.512	−0.778	0.500	−0.706
**2**-A2D${}_{A}$	−0.042	−0.747	0.505	−0.794	0.502	−0.704
**2**-A2D${}_{B}$	−0.039	−0.763	0.513	−0.781	0.511	−0.740

**Table 3:** Excitation energies (converted to wavelengths λ in nm) and oscillator strengths (f) for three lowest-lying singlet states of the studied *N*-oxides: isolated molecule vs. microsolvation.

System	S${}_{1}$ State		S${}_{2}$ State		S${}_{3}$ State	
	λ	f	λ	f	λ	f
isolated **1**	261.12	0.0804	238.77	0.0016	224.75	0.0293
**1**-A2	257.03	0.0707	228.43	0.0015	221.66	0.0268
**1**-AD	254.11	0.0617	219.77	0.0100	218.95	0.0060
**1**-A2D2	251.52	0.0570	218.92	0.0208	216.88	0.0018
isolated **2**	276.60	0.2321	266.75	0.0137	238.22	0.0018
**2**-A2	271.96	0.2174	264.75	0.0102	237.08	0.0018
**2**-A2D${}_{A}$	269.13	0.2043	263.34	0.0098	235.58	0.0041
**2**-A2D${}_{B}$	269.35	0.1992	261.93	0.0266	235.89	0.0121

## Data Availability

The data presented in the current study are available in the article and in the associated Supplementary Materials.

## Supplementary Materials

The following supporting information can be downloaded. Figure S1. The structures of the investigated *N*-oxides with atoms numbering scheme. BP indicates the bridged protons while the dotted line—the presence of intramolecular hydrogen bond. Coloring code: oxygen—red, nitrogen—blue, carbon—grey and hydrogen—-white. Figure S2. The overlap of the X-ray (yellow) and optimized (red) structures of the dimers used in the SAPT study: (a) dimer 1 of the compound 1, (b) dimer 2 of the compound 1 and (c) dimer 1 of the compound 2. Figure S3. Microsolvation models of the compound **1** obtained at the ωB97XD/6-311++G(d,p) level of theory. Figure S4. Microsolvation models of the compound **2** obtained at the ωB97XD/6-311++G(d,p) level of theory. Table S1. Interatomic distances of atoms involved in the intramolecular hydrogen bonds in compound **1** (isolated molecule). Comparison between experimental (X-ray) [1] and computed at the DFT level of theory data. The 6-311++G(d,p) basis set was applied during the simulations. The data were obtained in the gas phase and with a solvent reaction field using an IEF-PCM model and water as a solvent. The interatomic distances are given in Å while valence angle is given in [°]. Table S2. Interatomic distances of atoms involved in the intramolecular hydrogen bonds in compound **1** (monohydrate). Comparison between experimental (X-ray) [1] and computed at DFT level of theory data. The 6-311++G(d,p) basis set was applied during the simulations. The data were obtained in the gas phase and with solvent reaction field using an IEF-PCM model and water as a solvent. The interatomic distances are given in Å while valence angle is given in [°]. Table S3. Interatomic distances of atoms involved in the intramolecular hydrogen bonds in compound **2**. Comparison between experimental (X-ray) [2] and computed at DFT level of theory data. The 6-311++G(d,p) basis set was applied during the simulations. The data were obtained in the gas phase and with solvent reaction field using an IEF-PCM model and water as a solvent. The interatomic distances are given in Å while valence angle is given in [°]. Table S4. The energy of conformers of **1** and **2** computed at the B3LYP/6-311++G(d,p) and ωB97XD/6-311++G(d,p) levels of theory. The values of energy are given in kcal/mol. Table S5. Metric parameters of the intra- and intermolecular hydrogen bonds obtained as a result of microsolvation of the compound **1**. The interatomic distances are given in Å while valence angle is given in [°]. Table S6. Metric parameters of the intra- and intermolecular hydrogen bonds obtained as a result of microsolvation of the compound **2** (B3LYP/6-311++G(d,p) level of theory). The interatomic distances are given in Å while valence angle is given in [°]. Table S7. Metric parameters of the intra- and intermolecular hydrogen bonds obtained as a result of microsolvation of the compound **2** (ωB97XD/6-311++G(d,p) level of theory). The interatomic distances are given in Å while valence angle is given in [°]. MICROSOLVATION MODELS OF THE COMPOUND **1**. Sets of coordinates obtained at the B3LYP/6-311++G(d,p) level of theory. Sets of coordinates obtained at the ωB97XD/6-311++G(d,p) level of theory. MICROSOLVATION MODELS OF THE COMPOUND **2**. Sets of coordinates obtained at the B3LYP/6-311++G(d,p) level of theory. Sets of coordinates obtained at the ωB97XD/6-311++G(d,p) level of theory.

## Abbreviations

The following abbreviations are used in this manuscript:

DFT	Density Functional Theory
PES	Potential Energy Surface
PCM	Polarizable Continuum Model
TD-DFT	Time-Dependent Density Functional Theory
CCDC	Cambridge Crystallographic Data Centre
FT-IR	Fourier-Transform Infrared spectroscopy
NMR	Nuclear Magnetic Resonance
NBO	Natural Bond Orbital
SAPT	Symmetry-Adapted Perturbation Theory
BSSE	Basis Set Superposition Error
