# Supplementary material for: Exploring Intra- and Intermolecular Interactions in Selected N-Oxides—The Role of Hydrogen Bonds
Source: Molecules. 2022 Jan 25;27(3):792. doi: 10.3390/molecules27030792 (PMC8846293; doi:10.3390/molecules27030792)
Supplement: Supplementary file 1 [file molecules-27-00792-s001.zip › molecules-1564408-supplementary.pdf]

# SUPPLEMENTARY MATERIAL

# Exploring intra- and intermolecular interactions in selected *N*-oxides - the role of hydrogen bonds

Aneta Jezierska[1*], Jarosław J. Panek[1], Kacper Błaziak[2,3], Kamil Raczyński[1] and Aleksander Koll[4]

[1]University of Wrocław, Faculty of Chemistry, ul. F. Joliot-Curie 14, 50-383 Wrocław, Poland

[2]University of Warsaw, Faculty of Chemistry, ul. Pasteura 1, 01-224 Warsaw, Poland

[3] University of Warsaw, Biological and Chemical Research Center, Żwirki i Wigury 101, 01-224 Warsaw, Poland

[4]Non-public School of Medicine, ul. Nowowiejska 69, 50-340 Wrocław, Poland

Correspondence should be addressed to: aneta.jezierska@chem.uni.wroc.pl; Tel.: +48 71 3757 224; fax: +48 71 3282 348

**Table of content:**

I. **Figure S1.** The structures of the investigated *N*-oxides with atoms numbering scheme. BP indicates the bridged protons while the dotted line – the presence of intramolecular hydrogen bond. Coloring code: oxygen – red, nitrogen – blue, carbon – grey, hydrogen – white and bromine – pink.

II. **Table S1.** Interatomic distances of atoms involved in the intramolecular hydrogen bonds in **compound 1 (isolated molecule)**. Comparison between experimental (X-ray) [1] and computed at the DFT level of theory data. The 6-311++G(d,p) basis set was applied during the simulations. The data was obtained in the gas phase and with solvent reaction field using IEF-PCM model and water as a solvent. The interatomic distances are given in Å while valence angle is given in [°].

III. **Table S2.** Interatomic distances of atoms involved in the intramolecular hydrogen bonds in **compound 1 (monohydrate)**. Comparison between experimental (X-ray) [1] and computed at DFT level of theory data. The 6-311++G(d,p) basis set was applied during the simulations. The data was obtained in the gas phase and with solvent reaction field using IEF-PCM model and water as a solvent. The interatomic distances are given in Å while valence angle is given in [°].

**IV.** **Table S3.** Interatomic distances of atoms involved in the intramolecular hydrogen bonds in **compound 2**. Comparison between experimental (X-ray) [2] and computed at DFT level of theory data. The 6-311++G(d,p) basis set was applied during the simulations. The data was obtained in the gas phase and with solvent reaction field using IEF-PCM model and water as a solvent. The interatomic distances are given in Å while valence angle is given in [°].

**V.** **Table S4.** The energy of conformers of **1** and **2** computed at the B3LYP/6-311++G(d,p) and ωB97XD/6-311++G(d,p) levels of theory. The values of energy are given in kcal/mol.

**VI.** **Figure S2.** The overlap of the X-ray (yellow) and optimized (red) structures of the dimers used in the SAPT study: (a) dimer 1 of the compound **1**, (b) dimer 2 of the compound **1** and (c) dimer 1 of the compound **2**.

**VII.** **Figure S3.** Microsolvation models of the compound **1** obtained at the ωB97XD/6-311++G(d,p) level of theory.

**VIII.** **Table S5**. Metric parameters of the intra- and intermolecular hydrogen bonds obtained as a result of microsolvation of the compound **1**. The interatomic distances are given in Å while valence angle is given in [°].

**IX.** **MICROSOLVATION MODELS OF THE COMPOUND 1.**

Sets of coordinates obtained at the B3LYP/6-311++G(d,p) level of theory.

Sets of coordinates obtained at the ωB97XD/6-311++G(d,p) level of theory.

**X.** **Figure S4.** Microsolvation models of the compound **2** obtained at the ωB97XD/6-311++G(d,p) level of theory.

**XI.** **Table S6.** Metric parameters of the intra- and intermolecular hydrogen bonds obtained as a result of microsolvation of the compound **2** (B3LYP/6-311++G(d,p) level of theory). The interatomic distances are given in Å while valence angle is given in [°].

**XII.** **Table S7.** Metric parameters of the intra- and intermolecular hydrogen bonds obtained as a result of microsolvation of the compound **2** (ωB97XD/6-311++G(d,p) level of theory). The interatomic distances are given in Å while valence angle is given in [°].

**XIII.** **MICROSOLVATION MODELS OF THE COMPOUND 2.**

Sets of coordinates obtained at the B3LYP/6-311++G(d,p) level of theory.

Sets of coordinates obtained at the ωB97XD/6-311++G(d,p) level of theory.

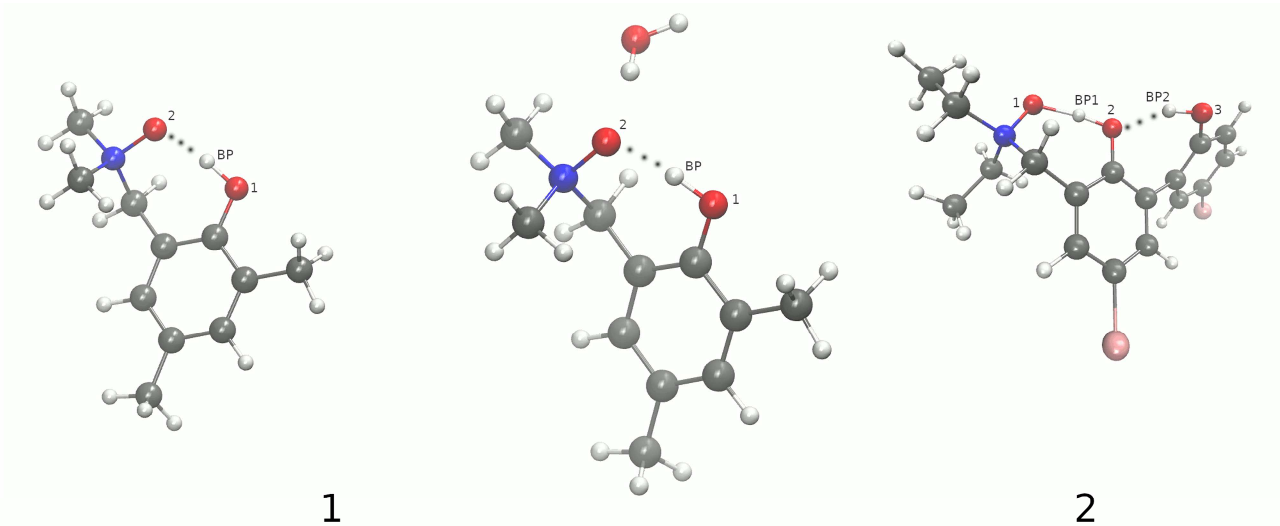


1 2

**Figure S1.** The structures of the investigated *N*-oxides with atoms numbering scheme. BP indicates the bridged protons while the dotted line – the presence of intramolecular hydrogen bond. Coloring code: oxygen – red, nitrogen – blue, carbon – grey, hydrogen – white and bromine – pink.

**Table S1.** Interatomic distances of atoms involved in the intramolecular hydrogen bonds in **compound 1 (isolated molecule)**. Comparison between experimental (X-ray) [1] and computed at the DFT level of theory data. The 6-311++G(d,p) basis set was applied during the simulations. The data was obtained in the gas phase and with solvent reaction field using IEF-PCM model and water as a solvent. The interatomic distances are given in Å while valence angle is given in [°].

| **Metric parameters** | **Exp. X-ray [1]** | **Gas phase** | | | | **Polarizable Continuum Model** | | | |
|---|---|---|---|---|---|---|---|---|---|
| | | **B3LYP** | **CAM-B3LYP** | **M05-2X** | **ωB97XD** | **B3LYP** | **CAM-B3LYP** | **M05-2X** | **ωB97XD** |
| O1...O2 | 2.541 | 2.5564 | 2.5427 | 2.5161 | 2.5608 | 2.5347 | 2.5214 | 2.4854 | 2.5407 |
| O1-HBP | 1.135 | 1.0171 | 1.0147 | 1.0246 | 1.0082 | 1.0239 | 1.0213 | 1.0386 | 1.0132 |
| HBP...O2 | 1.458 | 1.5514 | 1.5421 | 1.5049 | 1.5664 | 1.5193 | 1.5101 | 1.4557 | 1.5374 |
| <O1HBPO2 | 156.85 | 168.63 | 167.71 | 167.92 | 167.89 | 170.51 | 169.64 | 170.15 | 169.71 |

*the experimental data was recorded for the monohydrated form of the compound **1**. Therefore the X-ray values serve only as references for our quantum-mechanical simulations for the isolated molecule.

**the Cambridge Crystallographic Data Center (CCDC) identifier for the **compound 1** is: SEHBEM with the database number: 593412.

**Table S2.** Interatomic distances of atoms involved in the intramolecular hydrogen bonds in **compound 1 (monohydrate)**. Comparison between experimental (X-ray) [1] and computed at DFT level of theory data. The 6-311++G(d,p) basis set was applied during the simulations. The data was obtained in the gas phase and with solvent reaction field using IEF-PCM model and water as a solvent. The interatomic distances are given in Å while valence angle is given in [°].

| Metric parameters | Exp. X-ray [1] | Gas phase | | | | Polarizable Continuum Model | | | |
|---|---|---|---|---|---|---|---|---|---|
| | | B3LYP | CAM-B3LYP | M05-2X | ωB97XD | B3LYP | CAM-B3LYP | M05-2X | ωB97XD |
| **Intramolecular hydrogen bond** | | | | | | | | | |
| **O1...O2** | 2.541 | 2.5882 | 2.5748 | 2.5541 | 2.5905 | 2.5694 | 2.5553 | 2.5324 | 2.5787 |
| **O1-HBP** | 1.135 | 1.0073 | 1.0047 | 1.0099 | 0.9991 | 1.0097 | 1.0068 | 1.0135 | 1.0013 |
| **HBP...O2** | 1.458 | 1.5894 | 1.5794 | 1.5533 | 1.6013 | 1.5717 | 1.5625 | 1.5336 | 1.5874 |
| **<O1HBPO2** | 156.85 | 170.44 | 170.03 | 170.14 | 169.73 | 168.68 | 167.73 | 167.41 | 169.67 |
| **Intermolecular hydrogen bond** | | | | | | | | | |
| **O2...O** | 2.718 | 2.7777 | 2.7348 | 2.7614 | 2.7498 | 2.7275 | 2.6946 | 2.7189 | 2.7307 |
| **O-H** | 0.964 | 0.9816 | 0.9815 | 0.9755 | 0.9781 | 0.9866 | 0.9866 | 0.9808 | 0.9814 |
| **O2...H** | 1.755 | 1.8227 | 1.7902 | 1.8317 | 1.8084 | 1.7427 | 1.7085 | 1.7403 | 1.7500 |
| **O2HO** | 177.46 | 163.44 | 160.42 | 158.22 | 160.47 | 175.64 | 177.66 | 175.10 | 177.32 |

**the Cambridge Crystallographic Data Center (CCDC) identifier for the **compound 1** is: SEHBEM with the database number: 593412.

**Table S3.** Interatomic distances of atoms involved in the intramolecular hydrogen bonds in **compound 2**. Comparison between experimental (X-ray) [2] and computed at DFT level of theory data. The 6-311++G(d,p) basis set was applied during the simulations. The data was obtained in the gas phase and with solvent reaction field using IEF-PCM model and water as a solvent. The interatomic distances are given in Å while valence angle is given in [°].

| Metric parameters | Exp. X-ray [2] | Gas phase | | | | Polarizable Continuum Model | | | |
|---|---|---|---|---|---|---|---|---|---|
| | | B3LYP | CAM-B3LYP | M05-2X | ωB97XD | B3LYP | CAM-B3LYP | M05-2X | ωB97XD |
| **O2...O1** | 2.419 | 2.4559 | 2.4459 | 2.4215 | 2.4636 | 2.4695* | 2.4466* | 2.4291* | 2.4729* |
| **O2-HBP1** | 1.215 | 1.0663 | 1.0626 | 1.0946 | 1.0479 | 1.4152* | 1.3865* | 1.3495* | 1.4344* |
| **HBP1...O1** | 1.205 | 1.3952 | 1.3902 | 1.3318 | 1.4228 | 1.0565* | 1.0627* | 1.0820* | 1.0425* |
| **<O1HBP1O2** | 178.71 | 172.28 | 171.36 | 172.65 | 171.21 | 175.15* | 174.69* | 174.83* | 173.36* |
| **O2...O3** | 2.529 | 2.6363 | 2.6176 | 2.6309 | 2.6436 | 2.5444 | 2.5306 | 2.5294 | 2.5509 |
| **O3-HBP2** | 1.284 | 0.9791 | 0.9779 | 0.9755 | 0.9734 | 1.0025 | 1.0008 | 1.0005 | 0.9953 |
| **HBP2...O2** | 1.245 | 1.7195 | 1.7033 | 1.7201 | 1.7380 | 1.5741 | 1.5637 | 1.5651 | 1.5907 |
| **<O3HBP2O2** | 179.60 | 154.34 | 154.01 | 153.81 | 153.22 | 161.39 | 160.85 | 160.23 | 160.59 |

*indicates that the bridged proton moved to the acceptor side

**the Cambridge Crystallographic Data Center (CCDC) identifier for the **compound 2** is: WUKMOE with the database number: 178615.

**Table S4.** The energy of conformers of **1** and **2** computed at the B3LYP/6-311++G(d,p) and ωB97XD/6-311++G(d,p) levels of theory. The values of energy are given in kcal/mol.

| Level of theory | B3LYP/6-311++G(d,p) | | ωB97XD/6-311++G(d,p) | |
|---|---|---|---|---|
| | Gas phase | PCM | Gas phase | PCM |
| **Compound 1*** | | | | |
| **A** | -398299.043 | -398309.856 | -398168,700 | -398179,869 |
| **B** | --- | -398297.785 | --- | -398167,881 |
| **Compound 1 (monohydrate)*** | | | | |
| **A** | -446286.714 | -446299.528 | -446142.652 | -446154.563 |
| **B** | -446273.171 | -446289.713 | --- | -446145.995 |
| **Compound 2** | | | | |
| **A** | -3820383.622 | -3820396.998 (proton transfer to the N→O moiety) | -3820229.650 | -3820243.133 (proton transfer to the N→O moiety) |
| **B** | -3820377.413 | -3820391.784 | -3820223.445 | -3820238.145 |
| **C** | -3820362.620 | -3820380.499 | -3820208.319 | -3820226.876 |

*for the isolated molecule of **1** there was not found an open form in the gas phase for both functionals. The same holds for monohydrate form of the computed **1** computed at the ωB97XD/6-311++G(d,p) level of theory in the gas phase.

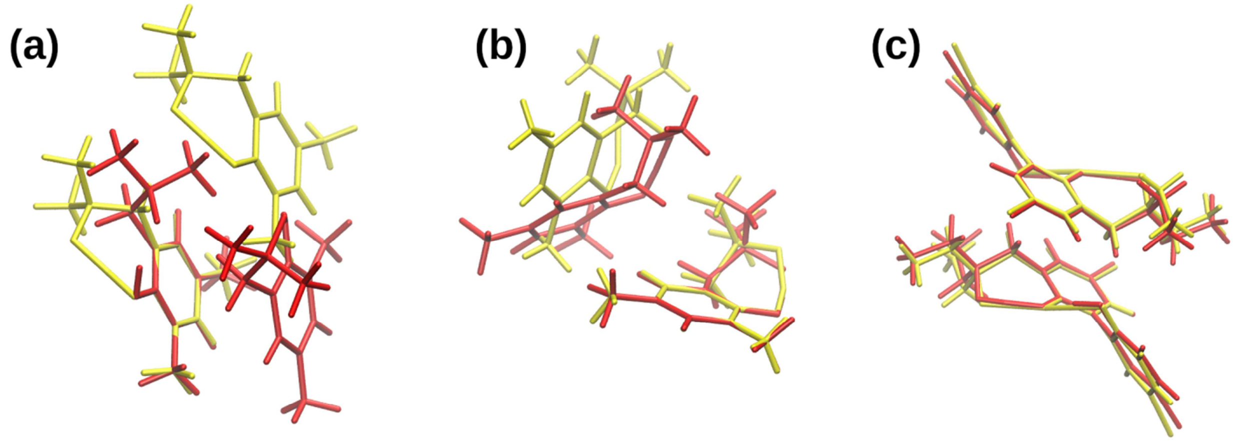


**Figure S2.** The overlap of the X-ray (yellow) and optimized (red) structures of the dimers used in the SAPT study: (a) dimer 1 of the compound **1**, (b) dimer 2 of the compound **1** and (c) dimer 1 of the compound **2**.

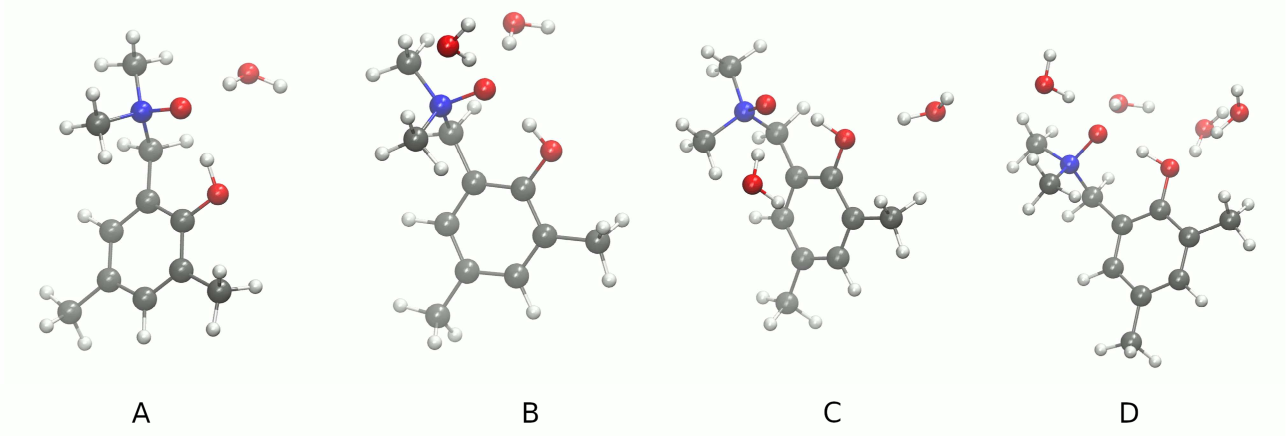


**Figure S3.** Microsolvation models of the compound **1** obtained at the ωB97XD/6-311++G(d,p) level of theory.

**Table S5**. Metric parameters of the intra- and intermolecular hydrogen bonds obtained as a result of microsolvation of the compound **1**. The interatomic distances are given in Å while valence angle is given in [°].

| | | | | | | | | |
|---|---|---|---|---|---|---|---|---|
| | **Compound 1** | | | | | | | |
| | **B3LYP/6-311++G(d,p)** | | | | **ωB97XD/6-311++G(d,p)** | | | |
| | **Model A** | | | | | | | |
| | **Intramolecular hydrogen bond (interatomic distances)** | | | | | | | |
| | O1...O2 | O1-HBP | HBP...O2 | <O1HBPO2 | O1...O2 | O1-HBP | HBP...O2 | <O1HBPO2 |
| | 2.5882 | 1.0073 | 1.5894 | 170.44 | 2.5905 | 0.9991 | 1.6013 | 169.73 |
| | **Intermolecular hydrogen bond (interatomic distances)** | | | | | | | |
| | O2...O | O-H | O2...H | <O2HO | O2...O | O-H | O2...H | <O2HO |
| | 2.7777 | 0.9816 | 1.8227 | 163.44 | 2.7498 | 0.9781 | 1.8084 | 160.47 |
| | **Model B** | | | | | | | |
| | **Intramolecular hydrogen bond (interatomic distances)** | | | | | | | |
| | O1...O2 | O1-HBP | HBP...O2 | <O1HBPO2 | O1...O2 | O1-HBP | HBP...O2 | <O1HBPO2 |
| | 2.6113 | 0.9994 | 1.6300 | 170.14 | 2.6089 | 0.9921 | 1.6284 | 168.91 |
| | **Intermolecular hydrogen bonds (interatomic distances)** | | | | | | | |
| | O2...O | O-H | O2...H | <O2HO | O2...O | O-H | O2...H | <O2HO |
| $H_2O$ (1) | 2.7629 | 0.9804 | 1.8273 | 158.49 | 2.7364 | 0.9768 | 1.8071 | 157.78 |
| $H_2O$ (2) | 2.8057 | 0.9787 | 1.8592 | 161.83 | 2.7781 | 0.9749 | 1.8370 | 161.32 |

**Table S5 (Continuation).** Metric parameters of the intra- and intermolecular hydrogen bonds obtained as a result of microsolvation of the compound **1**. The interatomic distances are given in Å while valence angle is given in [°].

| **Compound 1** | | | | | | | | |
|---|---|---|---|---|---|---|---|---|
| | **B3LYP/6-311++G(d,p)** | | | | **ωB97XD/6-311++G(d,p)** | | | |
| | **Model C** | | | | | | | |
| | **Intramolecular hydrogen bond (interatomic distances)** | | | | | | | |
| | O1...O2 | O1-HBP | HBP...O2 | <O1HBPO2 | O1...O2 | O1-HBP | HBP...O2 | <O1HBPO2 |
| | 2.4734 | 1.0544 | 1.4261 | 171.23 | 2.5573 | 1.0040 | 1.5880 | 160.73 |
| | **Intermolecular hydrogen bonds (interatomic distances)** | | | | | | | |
| | O1...O | O-H | O1...H | <O1HO | O2...O | O-H | O2...H | <O2HO |
| $H_2O$ | 2.7676 | 0.9814 | 1.7995 | 168.26 | 2.8700 | 0.9722 | 1.9088 | 165.69 |
| | --- | --- | --- | --- | O1...O | O-H | O1...H | <O1HO |
| | --- | --- | --- | --- | 2.8307 | 0.9682 | 1.8668 | 173.32 |
| | **Model D** | | | | | | | |
| | **B3LYP/6-311++G(d,p)** | | | | **ωB97XD/6-311++G(d,p)** | | | |
| | **Intramolecular hydrogen bond (interatomic distances)** | | | | | | | |
| | O1...O2 | O1-HBP | HBP...O2 | <O1HBPO2 | O1...O2 | O1-HBP | HBP...O2 | <O1HBPO2 |
| | 2.5448 | 1.0206 | 1.5307 | 171.65 | 2.5328 | 1.0143 | 1.5267 | 170.58 |
| | **Intermolecular hydrogen bonds (interatomic distances)** | | | | | | | |
| | O2...O | O-H | O2...H | <O2HO | O2...O | O-H | O2...H | <O2HO |
| $H_2O$ (1) | 2.7604 | 0.9805 | 1.8211 | 159.37 | 2.7412 | 0.9759 | 1.8114 | 158.09 |
| $H_2O$ (2) | 2.9436 | 0.9692 | 2.0813 | 147.26 | 2.8951 | 0.9665 | 2.0071 | 151.79 |
| | O1...O | O-H | O1...H | <O1HO | O1...O | O-H | O1...H | <O1HO |
| $H_2O$ (1) | 2.7903 | 0.9792 | 1.8128 | 175.92 | 2.8290 | 0.9683 | 1.9147 | 156.47 |
| $H_2O$ (2) | --- | --- | --- | --- | 2.9572 | 0.9636 | 2.1026 | 146.93 |

## MICROSOLVATION MODELS OF THE COMPOUND 1

## Sets of coordinates obtained at the B3LYP/6-311++G(d,p) level of theory

### Compound 1

**Model A - monohydrated form**

34

scf done: -711.203686

| | | | |
|---|---|---|---|
| O | 16.387287 | 1.562442 | 1.413093 |
| O | 17.733176 | 2.005451 | 3.578938 |
| N | 17.074276 | 3.065476 | 4.195723 |
| C | 15.250822 | 2.261370 | 1.603347 |
| C | 15.173909 | 3.364542 | 2.474735 |
| C | 13.942928 | 4.019110 | 2.641267 |
| C | 12.799343 | 3.630304 | 1.953804 |
| C | 12.915976 | 2.548890 | 1.068650 |
| C | 14.108398 | 1.860460 | 0.877235 |
| C | 16.393874 | 3.946061 | 3.136386 |
| C | 18.088478 | 3.892966 | 4.930163 |
| C | 16.081107 | 2.509435 | 5.165402 |
| C | 11.481362 | 4.345423 | 2.135749 |
| C | 14.214358 | 0.698428 | -0.074515 |
| H | 16.988827 | 1.684023 | 2.211849 |
| H | 13.893228 | 4.872345 | 3.312839 |
| H | 12.040099 | 2.228349 | 0.510597 |
| H | 16.129216 | 4.882656 | 3.630640 |
| H | 17.197225 | 4.137075 | 2.422521 |
| H | 18.780123 | 4.308463 | 4.200623 |
| H | 18.624291 | 3.221716 | 5.597471 |
| H | 17.586787 | 4.682309 | 5.492355 |
| H | 15.387153 | 1.883622 | 4.612151 |
| H | 16.635964 | 1.909916 | 5.883189 |
| H | 15.547386 | 3.322558 | 5.660925 |
| H | 10.704216 | 3.666917 | 2.502329 |
| H | 11.121892 | 4.768632 | 1.192333 |
| H | 11.570649 | 5.164653 | 2.852804 |
| H | 14.946932 | 0.899420 | -0.861781 |

| | | | |
|---|---|---|---|
| H | 13.250806 | 0.489050 | -0.543232 |
| H | 14.557482 | -0.202231 | 0.442293 |
| O | 20.008108 | 3.181740 | 2.503488 |
| H | 19.280948 | 2.607789 | 2.828091 |
| H | 20.317376 | 2.780940 | 1.686637 |

**Model B**

37

scf done: -787.676848

| | | | |
|---|---|---|---|
| O | 16.350714 | 1.517713 | 1.375271 |
| O | 17.696746 | 1.874759 | 3.584199 |
| N | 17.048042 | 2.936876 | 4.227393 |
| C | 15.234474 | 2.252054 | 1.576499 |
| C | 15.187226 | 3.331235 | 2.478336 |
| C | 13.976874 | 4.021895 | 2.649958 |
| C | 12.828609 | 3.691384 | 1.939988 |
| C | 12.918122 | 2.631932 | 1.026218 |
| C | 14.088936 | 1.908596 | 0.828459 |
| C | 16.416871 | 3.855141 | 3.171052 |
| C | 18.071724 | 3.718585 | 5.002084 |
| C | 16.017746 | 2.384051 | 5.164167 |
| C | 11.532418 | 4.442473 | 2.132428 |
| C | 14.165184 | 0.768665 | -0.152614 |
| H | 16.942220 | 1.594388 | 2.177195 |
| H | 13.947796 | 4.856865 | 3.344955 |
| H | 12.038367 | 2.356474 | 0.450648 |
| H | 16.174851 | 4.787608 | 3.683446 |
| H | 17.238220 | 4.039188 | 2.476169 |
| H | 18.784644 | 4.133738 | 4.292893 |
| H | 18.569772 | 3.021465 | 5.672659 |
| H | 17.573317 | 4.510146 | 5.563398 |
| H | 15.323052 | 1.785805 | 4.581679 |
| H | 16.535194 | 1.763914 | 5.892614 |
| H | 15.491811 | 3.207779 | 5.649440 |
| H | 10.743290 | 3.787464 | 2.516027 |

| | | | |
|---|---|---|---|
| H | 11.171379 | 4.865526 | 1.189798 |
| H | 11.652395 | 5.265433 | 2.840574 |
| H | 14.912836 | 0.963562 | -0.927093 |
| H | 13.200148 | 0.605844 | -0.636260 |
| H | 14.469776 | -0.156906 | 0.343960 |
| H | 19.154754 | 2.577297 | 2.669204 |
| O | 19.796400 | 3.210696 | 2.288563 |
| H | 20.009951 | 2.876443 | 1.412893 |
| H | 18.385726 | 0.797781 | 4.889764 |
| O | 18.610221 | 0.488161 | 5.792516 |
| H | 19.243214 | -0.226806 | 5.687024 |

**Model C**

37

scf done: -787.676234

| | | | |
|---|---|---|---|
| O | 15.635515 | 0.765330 | 1.915398 |
| O | 16.856615 | 1.367923 | 3.980263 |
| N | 16.844051 | 2.753160 | 4.091964 |
| C | 14.684906 | 1.731577 | 1.871810 |
| C | 14.943293 | 3.017120 | 2.380445 |
| C | 13.924737 | 3.979034 | 2.353743 |
| C | 12.668695 | 3.704521 | 1.819933 |
| C | 12.453945 | 2.424082 | 1.293460 |
| C | 13.431724 | 1.432466 | 1.305502 |
| C | 16.333931 | 3.409002 | 2.804286 |
| C | 18.254453 | 3.207247 | 4.323455 |
| C | 15.981216 | 3.121138 | 5.256169 |
| C | 11.572524 | 4.743873 | 1.800479 |
| C | 13.179895 | 0.059046 | 0.740828 |
| H | 16.214633 | 0.952819 | 2.776393 |
| H | 14.132519 | 4.973640 | 2.739493 |
| H | 11.484349 | 2.187548 | 0.863004 |
| H | 16.387657 | 4.487787 | 2.965317 |
| H | 17.077845 | 3.134982 | 2.054251 |
| H | 18.833815 | 2.934649 | 3.443544 |

| | | | |
|---|---|---|---|
| H | 18.619383 | 2.675365 | 5.199201 |
| H | 18.278290 | 4.286961 | 4.486011 |
| H | 14.975722 | 2.765178 | 5.047723 |
| H | 16.383176 | 2.609039 | 6.127302 |
| H | 15.985227 | 4.203265 | 5.402097 |
| H | 10.727532 | 4.446488 | 2.430469 |
| H | 11.183460 | 4.896711 | 0.789106 |
| H | 11.933742 | 5.707736 | 2.166226 |
| H | 13.842105 | -0.149614 | -0.105179 |
| H | 12.147826 | -0.038651 | 0.398365 |
| H | 13.376710 | -0.713780 | 1.488907 |
| H | 18.696736 | 1.800480 | 0.643160 |
| O | 19.091153 | 2.618750 | 1.011101 |
| H | 19.516577 | 3.053242 | 0.266632 |
| H | 16.876740 | 0.465564 | 0.647464 |
| O | 17.674044 | 0.406197 | 0.078296 |
| H | 17.995521 | -0.494795 | 0.177279 |

**Model D**

43

scf done: -940.624219

| | | | |
|---|---|---|---|
| O | 15.903728 | 1.102981 | 1.793657 |
| O | 17.359271 | 1.661106 | 3.805113 |
| N | 16.921475 | 2.914034 | 4.256215 |
| C | 14.873904 | 1.989802 | 1.882799 |
| C | 15.016827 | 3.219153 | 2.548315 |
| C | 13.908278 | 4.074081 | 2.633143 |
| C | 12.680955 | 3.754205 | 2.061799 |
| C | 12.581451 | 2.530600 | 1.388388 |
| C | 13.647615 | 1.641171 | 1.286660 |
| C | 16.344250 | 3.701592 | 3.074460 |
| C | 18.107925 | 3.659558 | 4.802040 |
| C | 15.905993 | 2.717303 | 5.339985 |
| C | 11.499432 | 4.691014 | 2.146898 |
| C | 13.511880 | 0.325414 | 0.565172 |
| H | 16.542065 | 1.261317 | 2.574091 |

| | | | |
|---|---|---|---|
| H | 14.022206 | 5.025070 | 3.146171 |
| H | 11.634711 | 2.254753 | 0.931945 |
| H | 16.246561 | 4.733713 | 3.414333 |
| H | 17.137173 | 3.650195 | 2.325456 |
| H | 18.804820 | 3.825066 | 3.983163 |
| H | 18.557249 | 3.028813 | 5.566327 |
| H | 17.771669 | 4.605341 | 5.229515 |
| H | 15.083183 | 2.138465 | 4.930047 |
| H | 16.388556 | 2.170846 | 6.147130 |
| H | 15.550355 | 3.690743 | 5.681642 |
| H | 10.622101 | 4.190236 | 2.567450 |
| H | 11.215762 | 5.065661 | 1.158067 |
| H | 11.722717 | 5.555303 | 2.776138 |
| H | 14.158070 | 0.287052 | -0.317334 |
| H | 12.482692 | 0.167386 | 0.238002 |
| H | 13.804332 | -0.508181 | 1.209808 |
| H | 18.925702 | 2.122090 | 2.514511 |
| O | 19.388024 | 2.804462 | 2.004637 |
| H | 19.595726 | 2.396869 | 1.143758 |
| H | 17.984833 | 0.742456 | 5.247676 |
| O | 18.220327 | 0.546295 | 6.179069 |
| H | 18.838011 | -0.189245 | 6.154795 |
| H | 19.810830 | 1.973367 | -1.322295 |
| O | 19.676492 | 1.483694 | -0.506072 |
| H | 18.814589 | 1.028568 | -0.603098 |
| H | 16.702010 | 0.531994 | 0.269554 |
| O | 17.194580 | 0.236420 | -0.523380 |
| H | 17.173424 | -0.724893 | -0.500807 |

## Sets of coordinates obtained at the ωB97XD/6-311++G(d,p) level of theory

**Compound 1**

**Model A - monohydrated form**

34

scf done: -710.974108

| | | | |
|---|---|---|---|
| O | 16.416340 | 1.561935 | 1.409566 |
| O | 17.715482 | 1.950463 | 3.616781 |
| N | 17.069178 | 3.015260 | 4.208963 |
| C | 15.292941 | 2.270252 | 1.604643 |
| C | 15.232650 | 3.369349 | 2.471982 |
| C | 14.014840 | 4.034436 | 2.648516 |
| C | 12.866557 | 3.657787 | 1.971611 |
| C | 12.964790 | 2.579055 | 1.087851 |
| C | 14.145363 | 1.881201 | 0.890293 |
| C | 16.458792 | 3.914402 | 3.146616 |
| C | 18.069203 | 3.804716 | 4.982313 |
| C | 16.028467 | 2.486721 | 5.127535 |
| C | 11.557782 | 4.378683 | 2.166893 |
| C | 14.237141 | 0.716708 | -0.054166 |
| H | 16.996978 | 1.648568 | 2.217993 |
| H | 13.977332 | 4.885476 | 3.324642 |
| H | 12.079960 | 2.267568 | 0.537992 |
| H | 16.216129 | 4.861169 | 3.633916 |
| H | 17.279690 | 4.078771 | 2.445003 |
| H | 18.780363 | 4.233320 | 4.278120 |
| H | 18.581824 | 3.109269 | 5.643471 |
| H | 17.564388 | 4.586012 | 5.553239 |
| H | 15.353974 | 1.864258 | 4.545723 |
| H | 16.537246 | 1.887577 | 5.879461 |
| H | 15.481008 | 3.313132 | 5.585403 |
| H | 10.806645 | 3.720972 | 2.614817 |
| H | 11.155659 | 4.734327 | 1.214096 |
| H | 11.676807 | 5.244128 | 2.822565 |
| H | 14.960959 | 0.913977 | -0.849498 |
| H | 13.267977 | 0.505256 | -0.509926 |

| | | | |
|---|---|---|---|
| H | 14.585068 | -0.178312 | 0.468048 |
| O | 19.741548 | 3.300763 | 2.338933 |
| H | 19.136684 | 2.645509 | 2.740748 |
| H | 19.964237 | 2.957591 | 1.473957 |

**Model B**

37

scf done: -787.424895

| | | | |
|---|---|---|---|
| O | 16.404105 | 1.570174 | 1.331179 |
| O | 17.663550 | 1.859133 | 3.597610 |
| N | 17.045210 | 2.935513 | 4.214136 |
| C | 15.281429 | 2.277720 | 1.554432 |
| C | 15.229736 | 3.341412 | 2.464013 |
| C | 14.016166 | 4.007505 | 2.665453 |
| C | 12.866656 | 3.665227 | 1.972607 |
| C | 12.958350 | 2.621731 | 1.047106 |
| C | 14.134516 | 1.923773 | 0.823288 |
| C | 16.459426 | 3.854201 | 3.157435 |
| C | 18.070790 | 3.691119 | 4.992679 |
| C | 15.989093 | 2.426603 | 5.131326 |
| C | 11.562630 | 4.387465 | 2.192063 |
| C | 14.217961 | 0.796625 | -0.166377 |
| H | 16.967253 | 1.609223 | 2.147000 |
| H | 13.983982 | 4.831530 | 3.374186 |
| H | 12.072695 | 2.338448 | 0.483668 |
| H | 16.228406 | 4.795375 | 3.660121 |
| H | 17.287546 | 4.020129 | 2.465079 |
| H | 18.798012 | 4.091398 | 4.288045 |
| H | 18.547477 | 2.984457 | 5.669902 |
| H | 17.583531 | 4.494205 | 5.547704 |
| H | 15.303502 | 1.820816 | 4.544538 |
| H | 16.476989 | 1.820156 | 5.892181 |
| H | 15.460282 | 3.271485 | 5.576136 |
| H | 10.790866 | 3.706596 | 2.563261 |
| H | 11.191882 | 4.826320 | 1.261302 |

| | | | |
|---|---|---|---|
| H | 11.673715 | 5.194095 | 2.919965 |
| H | 14.948711 | 1.016890 | -0.949197 |
| H | 13.248762 | 0.617270 | -0.635440 |
| H | 14.550029 | -0.123681 | 0.320993 |
| H | 19.087372 | 2.543433 | 2.659987 |
| O | 19.691360 | 3.176114 | 2.229538 |
| H | 19.764860 | 2.885062 | 1.320581 |
| H | 18.301382 | 0.812344 | 4.925389 |
| O | 18.505239 | 0.535017 | 5.839563 |
| H | 19.139553 | -0.177661 | 5.774052 |

**Model C**

37

scf done: -787.417341

| | | | |
|---|---|---|---|
| O | 16.580729 | 1.744942 | 1.071822 |
| O | 17.368202 | 1.584912 | 3.499570 |
| N | 17.057584 | 2.780355 | 4.107502 |
| C | 15.381019 | 2.303386 | 1.364325 |
| C | 15.257372 | 3.244306 | 2.391038 |
| C | 13.999212 | 3.767733 | 2.690931 |
| C | 12.867670 | 3.384720 | 1.986039 |
| C | 13.030128 | 2.466833 | 0.945763 |
| C | 14.262224 | 1.918048 | 0.614215 |
| C | 16.479828 | 3.750176 | 3.095823 |
| C | 18.313051 | 3.354318 | 4.659525 |
| C | 16.087299 | 2.539301 | 5.212270 |
| C | 11.504522 | 3.930229 | 2.323455 |
| C | 14.425590 | 0.933399 | -0.509273 |
| H | 17.075224 | 1.647003 | 1.940037 |
| H | 13.909861 | 4.500668 | 3.488814 |
| H | 12.158270 | 2.162957 | 0.372171 |
| H | 16.264287 | 4.681064 | 3.624895 |
| H | 17.289162 | 3.926189 | 2.383996 |
| H | 19.005782 | 3.488733 | 3.831473 |
| H | 18.720613 | 2.628322 | 5.359335 |

| | | | |
|---|---|---|---|
| H | 18.108716 | 4.304553 | 5.156799 |
| H | 15.188615 | 2.098044 | 4.787476 |
| H | 16.548629 | 1.827840 | 5.893958 |
| H | 15.866421 | 3.480794 | 5.718751 |
| H | 10.865788 | 3.152202 | 2.752204 |
| H | 11.000965 | 4.315096 | 1.432749 |
| H | 11.571046 | 4.743582 | 3.049151 |
| H | 15.064456 | 1.346312 | -1.295267 |
| H | 13.458404 | 0.677066 | -0.944983 |
| H | 14.906402 | 0.016896 | -0.156556 |
| H | 17.330080 | 2.060069 | -0.608666 |
| O | 17.620139 | 2.270750 | -1.508086 |
| H | 18.064163 | 1.480666 | -1.815250 |
| H | 14.590217 | 0.163893 | 2.865777 |
| O | 15.055633 | -0.091358 | 3.664493 |
| H | 15.922963 | 0.341081 | 3.587621 |

**Model D**

43

scf done: -940.321227

| | | | |
|---|---|---|---|
| O | 16.172074 | 1.350472 | 1.551886 |
| O | 17.413468 | 1.719601 | 3.728504 |
| N | 16.968753 | 2.936857 | 4.223313 |
| C | 15.083857 | 2.149023 | 1.703917 |
| C | 15.147884 | 3.309096 | 2.481147 |
| C | 13.997458 | 4.088172 | 2.626190 |
| C | 12.804714 | 3.754881 | 2.002117 |
| C | 12.786599 | 2.605167 | 1.209786 |
| C | 13.899992 | 1.792890 | 1.046205 |
| C | 16.441024 | 3.784931 | 3.083262 |
| C | 18.124166 | 3.645222 | 4.850821 |
| C | 15.913803 | 2.686512 | 5.243352 |
| C | 11.567405 | 4.600287 | 2.157379 |
| C | 13.864414 | 0.553079 | 0.197900 |
| H | 16.734501 | 1.428945 | 2.392300 |

| | | | |
|---|---|---|---|
| H | 14.048776 | 4.990479 | 3.230033 |
| H | 11.865360 | 2.328739 | 0.703536 |
| H | 16.311595 | 4.793425 | 3.480123 |
| H | 17.261004 | 3.794766 | 2.360968 |
| H | 18.852596 | 3.852166 | 4.068176 |
| H | 18.542078 | 2.975083 | 5.600001 |
| H | 17.770599 | 4.568962 | 5.311445 |
| H | 15.123565 | 2.108737 | 4.770769 |
| H | 16.366859 | 2.119021 | 6.054084 |
| H | 15.523578 | 3.641281 | 5.600290 |
| H | 10.754663 | 4.029134 | 2.615267 |
| H | 11.210896 | 4.958561 | 1.187642 |
| H | 11.758722 | 5.472381 | 2.785957 |
| H | 14.605276 | 0.595951 | -0.604922 |
| H | 12.879538 | 0.418461 | -0.251962 |
| H | 14.094144 | -0.334967 | 0.793787 |
| H | 18.998789 | 2.193394 | 2.592408 |
| O | 19.515658 | 2.822158 | 2.071262 |
| H | 19.352587 | 2.560721 | 1.151758 |
| H | 17.993343 | 0.759049 | 5.150614 |
| O | 18.190644 | 0.552055 | 6.083717 |
| H | 18.896163 | -0.093756 | 6.076125 |
| H | 17.485419 | 2.038354 | 0.061003 |
| O | 18.340635 | 1.936853 | -0.371264 |
| H | 18.357340 | 0.989083 | -0.555350 |
| H | 16.682685 | -0.189535 | 0.535188 |
| O | 17.193110 | -0.695873 | -0.113459 |
| H | 17.436017 | -1.513950 | 0.319666 |

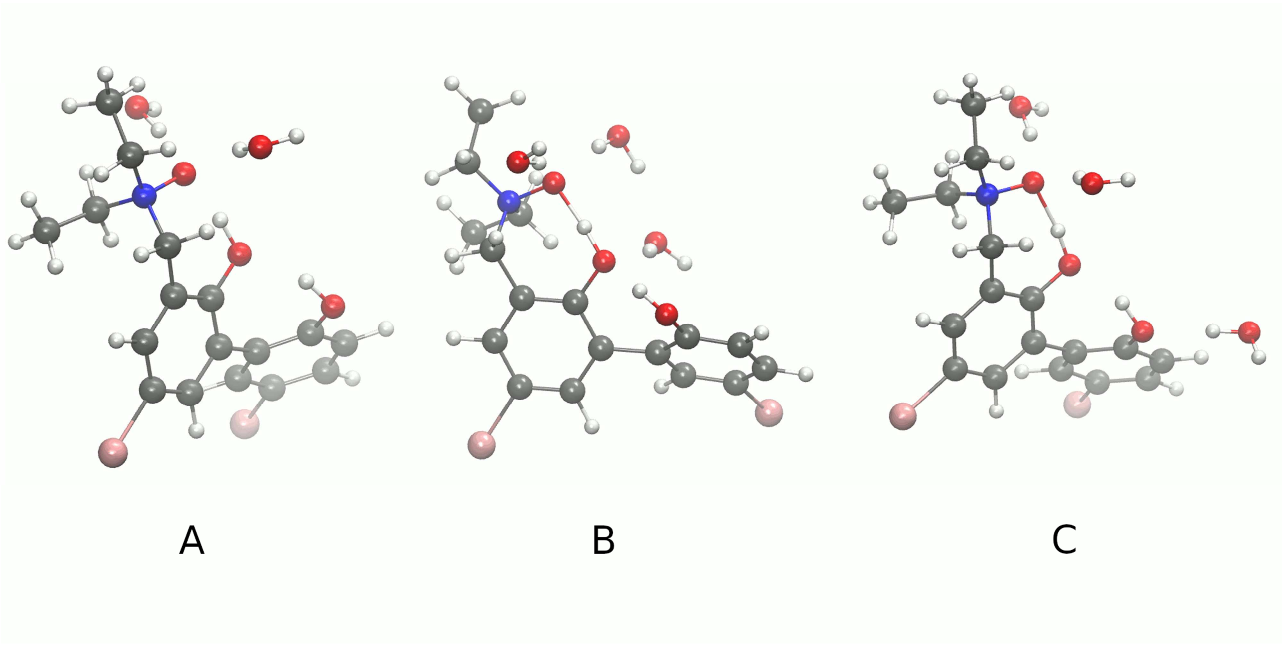


**Figure S4.** Microsolvation models of the compound **2** obtained at the ωB97XD/6-311++G(d,p) level of theory.

**Table S6.** Metric parameters of the intra- and intermolecular hydrogen bonds obtained as a result of microsolvation of the compound **2** (B3LYP/6-311++G(d,p) level of theory).. The interatomic distances are given in Å while valence angle is given in [°].

| **Compound 2** | | | | | | | | |
|---|---|---|---|---|---|---|---|---|
| | **B3LYP/6-311++G(d,p)** | | | | | | | |
| | **Model A** | | | | | | | |
| | **Intramolecular hydrogen bond (interatomic distances)** | | | | | | | |
| | O2...O1 | O2-HBP1 | HBP1...O2 | <O2HBP1O1 | O3...O2 | O3-HBP2 | HBP2...O2 | <O3HBP2O2 |
| | 2.5266 | 1.0231 | 1.5089 | 172.34 | 2.6616 | 0.9752 | 1.7595 | 152.24 |
| | **Intermolecular hydrogen bonds (interatomic distances)** | | | | | | | |
| | O1...O | O-H | O1...H | <O1HO | --- | --- | --- | --- |
| $H_2O$ (1) | 2.8106 | 0.9767 | 1.8511 | 166.70 | --- | --- | --- | --- |
| $H_2O$ (2) | 2.8175 | 0.9758 | 1.9020 | 155.17 | --- | --- | --- | --- |
| | **Model B** | | | | | | | |
| | **Intramolecular hydrogen bond (interatomic distances)** | | | | | | | |
| | O2…O1 | O2-HBP1 | HBP1…O2 | <O2HBP1O1 | O3…O2 | O3-HBP2 | HBP2…O2 | <O3HBP2O2 |
| | 2.4757 | 1.0516 | 1.4273 | 174.08 | 2.6820 | 0.9745 | 1.7893 | 150.72 |
| | **Intermolecular hydrogen bonds (interatomic distances)** | | | | | | | |
| | O1...O | O-H | O1...H | <O1HO | O2...O | O-H | O2...H | <O2HO |
| $H_2O$ (1) | 2.8071 | 0.9757 | 1.8972 | 154.04 | 2.9532 | 0.9696 | 2.0396 | 156.28 |
| $H_2O$ (2) | 2.9302 | 0.9693 | 1.9894 | 163.02 | --- | --- | --- | --- |

**Table S6 (Continuation).** Metric parameters of the intra- and intermolecular hydrogen bonds obtained as a result of microsolvation of the compound **2** (B3LYP/6-311++G(d,p) level of theory). The interatomic distances are given in Å while valence angle is given in [°].

| **Compound 2** | | | | | | | | |
|---|---|---|---|---|---|---|---|---|
| | **B3LYP/6-311++G(d,p)** | | | | | | | |
| | **Intramolecular hydrogen bond (interatomic distances)** | | | | | | | |
| | O2...O1 | O2-HBP1 | HBP1...O2 | <O2HBP1O1 | O3...O2 | O3-HBP2 | HBP2...O2 | <O3HBP2O2 |
| | 2.5147 | 1.0278 | 1.4919 | 172.66 | 2.6272 | 0.9796 | 1.7183 | 152.63 |
| | **Intermolecular hydrogen bonds (interatomic distances)** | | | | | | | |
| | O1...O | O-H | O1...H | <O1HO | O3...O | O-H | O3...H | <O3HO |
| $H_2O$ (1) | 2.8151 | 0.9762 | 1.8588 | 165.71 | 2.8813 | 0.9707 | 1.9182 | 171.21 |
| $H_2O$ (2) | 2.8215 | 0.9752 | 1.9118 | 154.12 | --- | --- | --- | --- |

**Table S7.** Metric parameters of the intra- and intermolecular hydrogen bonds obtained as a result of microsolvation of the compound **2** (ωB97XD/6-311++G(d,p) level of theory). The interatomic distances are given in Å while valence angle is given in [°].

| **Compound 2** | | | | | | | | |
|---|---|---|---|---|---|---|---|---|
| | **ωB97XD/6-311++G(d,p)** | | | | | | | |
| | **Model A** | | | | | | | |
| | **Intramolecular hydrogen bond (interatomic distances)** | | | | | | | |
| | O2...O1 | O2-HBP1 | HBP1...O2 | <O2HBP1O1 | O3...O2 | O3-HBP2 | HBP2...O2 | <O3HBP2O2 |
| | 2.5276 | 1.0122 | 1.5227 | 171.10 | 2.6666 | 0.9702 | 1.7750 | 151.22 |
| | **Intermolecular hydrogen bonds (interatomic distances)** | | | | | | | |
| | O1...O | O-H | O1...H | <O1HO | --- | --- | --- | --- |
| $H_2O$ (1) | 2.7723 | 0.9727 | 1.8613 | 154.72 | --- | --- | --- | --- |
| $H_2O$ (2) | 2.7745 | 0.9737 | 1.8193 | 166.16 | --- | --- | --- | --- |
| | **Model B** | | | | | | | |
| | **Intramolecular hydrogen bond (interatomic distances)** | | | | | | | |
| | O2...O1 | O2-HBP1 | HBP1...O2 | <O2HBP1O1 | O3...O2 | O3-HBP2 | HBP2...O2 | <O3HBP2O2 |
| | 2.4686 | 1.0393 | 1.4350 | 172.15 | 2.6833 | 0.9696 | 1.8068 | 148.76 |
| | **Intermolecular hydrogen bonds (interatomic distances)** | | | | | | | |
| | O1...O | O-H | O1...H | <O1HO | O2...O | O-H | O2...H | <O2HO |
| $H_2O$ (1) | 2.7619 | 0.9730 | 1.8548 | 153.89 | 2.8799 | 0.9661 | 1.9907 | 152.04 |
| $H_2O$ (2) | 2.8809 | 0.9666 | 1.9434 | 162.78 | --- | --- | --- | --- |

**Table S7 (Continuation).** Metric parameters of the intra- and intermolecular hydrogen bonds obtained as a result of microsolvation of the compound **2** (ωB97XD/6-311++G(d,p) level of theory). The interatomic distances are given in Å while valence angle is given in [°].

| **Compound 2** | | | | | | | | |
|---|---|---|---|---|---|---|---|---|
| | **ωB97XD/6-311++G(d,p)** | | | | | | | |
| | **Model C** | | | | | | | |
| | **Intramolecular hydrogen bond (interatomic distances)** | | | | | | | |
| | O2...O1 | O2-HBP1 | HBP1...O2 | <O2HBP1O1 | O3...O2 | O3-HBP2 | HBP2...O2 | <O3HBP2O2 |
| | 2.5170 | 1.0159 | 1.5080 | 171.39 | 2.6285 | 0.9743 | 1.7307 | 151.52 |
| | **Intermolecular hydrogen bonds (interatomic distances)** | | | | | | | |
| | O1...O | O-H | O1...H | <O1HO | O3...O2 | O3-HBP2 | HBP2...O2 | <O3HBP2O2 |
| $H_2O$ (1) | 2.7750 | 0.9724 | 1.8693 | 153.77 | 2.8524 | 0.9670 | 1.8983 | 168.45 |
| $H_2O$ (2) | 2.7768 | 0.9733 | 1.8237 | 165.54 | | | | |

## MICROSOLVATION MODELS OF THE COMPOUND 2

## Sets of coordinates obtained at the B3LYP/6-311++G(d,p) level of theory

**Compound 2**

**Model A**

48

scf done: -6241.115307

| | | | |
|---|---|---|---|
| Br | 3.957661 | 2.217929 | -4.649697 |
| Br | 6.575341 | 7.256155 | 0.406954 |
| O | 9.112137 | 1.189110 | -1.625247 |
| O | 10.263554 | 3.428306 | -2.488018 |
| O | 12.322647 | 4.746570 | -3.125037 |
| C | 7.466582 | 2.808989 | -2.420823 |
| C | 7.971200 | 1.495071 | -2.297952 |
| C | 7.253591 | 0.420986 | -2.834423 |
| H | 7.661297 | -0.574219 | -2.704912 |
| C | 6.060367 | 0.614491 | -3.515208 |
| H | 5.520126 | -0.227598 | -3.927941 |
| C | 5.583290 | 1.912460 | -3.672363 |
| C | 6.269251 | 2.990319 | -3.136509 |
| H | 5.883344 | 3.991905 | -3.275089 |
| C | 8.078230 | 4.003432 | -1.771949 |
| C | 9.457513 | 4.296698 | -1.836081 |
| C | 9.978061 | 5.459064 | -1.229771 |
| C | 9.106893 | 6.337705 | -0.578628 |
| H | 9.495663 | 7.225113 | -0.094347 |
| C | 7.750955 | 6.053383 | -0.515387 |
| C | 7.241279 | 4.899777 | -1.093368 |
| H | 6.185725 | 4.677249 | -1.011144 |
| C | 11.461222 | 5.729456 | -1.135062 |
| H | 11.996370 | 4.886877 | -0.693355 |
| H | 11.626219 | 6.607972 | -0.512046 |
| N | 12.207437 | 5.972437 | -2.453478 |
| C | 13.606248 | 6.464775 | -2.098107 |
| H | 13.478452 | 7.384450 | -1.524586 |

| | | | |
|---|---|---|---|
| H | 14.012495 | 5.696929 | -1.441053 |
| C | 14.511720 | 6.677185 | -3.298808 |
| H | 15.512754 | 6.906209 | -2.924902 |
| H | 14.194490 | 7.509834 | -3.929052 |
| H | 14.575361 | 5.781474 | -3.914542 |
| C | 11.471254 | 6.932467 | -3.371851 |
| H | 10.493764 | 6.478078 | -3.524604 |
| H | 12.005268 | 6.886501 | -4.318518 |
| C | 11.340444 | 8.361705 | -2.862924 |
| H | 10.748218 | 8.920670 | -3.591523 |
| H | 12.303872 | 8.866692 | -2.773347 |
| H | 10.817261 | 8.426093 | -1.907529 |
| H | 9.771019 | 1.887752 | -1.795125 |
| H | 11.131454 | 3.891672 | -2.768706 |
| H | 13.484195 | 3.620294 | -2.125051 |
| O | 13.997355 | 3.351081 | -1.340020 |
| H | 14.190657 | 2.415208 | -1.443507 |
| H | 12.627428 | 4.831560 | -4.948897 |
| O | 12.835776 | 5.093080 | -5.866576 |
| H | 12.431834 | 4.427564 | -6.430284 |

**Model B**

51

scf done: -6317.585502

| | | | |
|---|---|---|---|
| Br | -5.520416 | -0.854302 | -1.143819 |
| Br | -0.291065 | 4.585950 | 0.036164 |
| O | -0.932308 | -1.687540 | 2.732546 |
| O | 0.909335 | -1.297978 | 0.822178 |
| O | 3.252512 | -1.739183 | 0.155948 |
| C | -1.821653 | -0.477771 | 0.806302 |
| C | -1.915166 | -1.455067 | 1.822613 |
| C | -3.089486 | -2.202109 | 1.964504 |
| H | -3.133252 | -2.928747 | 2.766702 |
| C | -4.167435 | -2.024600 | 1.109254 |
| H | -5.067888 | -2.612535 | 1.231282 |

| | | | |
|---|---|---|---|
| C | -4.061784 | -1.089825 | 0.082738 |
| C | -2.913602 | -0.328747 | -0.068222 |
| H | -2.848878 | 0.389547 | -0.875221 |
| C | -0.671745 | 0.459191 | 0.658187 |
| C | 0.671859 | 0.029067 | 0.664530 |
| C | 1.726165 | 0.951371 | 0.500220 |
| C | 1.420863 | 2.301078 | 0.299666 |
| H | 2.220152 | 3.022319 | 0.180419 |
| C | 0.100438 | 2.726141 | 0.287894 |
| C | -0.935260 | 1.822770 | 0.473758 |
| H | -1.959549 | 2.170715 | 0.490894 |
| C | 3.185664 | 0.584473 | 0.656538 |
| H | 3.396800 | 0.190037 | 1.652325 |
| H | 3.792950 | 1.476315 | 0.504615 |
| N | 3.735833 | -0.495750 | -0.279238 |
| C | 5.251894 | -0.502568 | -0.130235 |
| H | 5.602550 | 0.486240 | -0.430038 |
| H | 5.426140 | -0.628126 | 0.937824 |
| C | 5.939538 | -1.603934 | -0.918381 |
| H | 7.000265 | -1.592290 | -0.656076 |
| H | 5.862933 | -1.466582 | -1.998228 |
| H | 5.536801 | -2.582252 | -0.659908 |
| C | 3.295018 | -0.303457 | -1.721703 |
| H | 2.207118 | -0.309427 | -1.689920 |
| H | 3.602323 | -1.210744 | -2.237273 |
| C | 3.817035 | 0.953320 | -2.403703 |
| H | 3.380393 | 0.991371 | -3.404568 |
| H | 4.901900 | 0.949426 | -2.524239 |
| H | 3.520901 | 1.869385 | -1.889221 |
| H | -0.068723 | -1.603553 | 2.289025 |
| H | 1.904745 | -1.533914 | 0.578597 |
| H | 3.972899 | -2.107793 | 1.871860 |
| O | 4.426065 | -1.870055 | 2.702633 |
| H | 4.296056 | -2.604219 | 3.309341 |
| H | 3.070457 | -3.162815 | -1.221731 |

| | | | |
|---|---|---|---|
| O | 2.848432 | -3.645110 | -2.032724 |
| H | 1.884177 | -3.558433 | -2.079589 |
| H | 0.099988 | -2.396292 | -0.693980 |
| O | 0.056438 | -2.792457 | -1.577901 |
| H | -0.828118 | -3.162493 | -1.655500 |

**Model C**

51

scf done: -6317.582662

| | | | |
|---|---|---|---|
| Br | -5.458306 | -0.189571 | -1.728928 |
| Br | -0.088311 | 4.504577 | 0.954862 |
| O | -1.070537 | -2.221494 | 1.934455 |
| O | 0.864817 | -1.440385 | 0.338757 |
| O | 3.192684 | -1.717432 | -0.571155 |
| C | -1.844165 | -0.511869 | 0.378899 |
| C | -2.019644 | -1.721311 | 1.082824 |
| C | -3.214532 | -2.435983 | 0.974647 |
| H | -3.317332 | -3.354704 | 1.540330 |
| C | -4.245597 | -1.986970 | 0.160479 |
| H | -5.165792 | -2.550867 | 0.080862 |
| C | -4.065326 | -0.812060 | -0.562809 |
| C | -2.889615 | -0.085629 | -0.459597 |
| H | -2.767065 | 0.819813 | -1.039451 |
| C | -0.653099 | 0.372728 | 0.521097 |
| C | 0.673013 | -0.108678 | 0.478741 |
| C | 1.766677 | 0.775188 | 0.586687 |
| C | 1.525131 | 2.146312 | 0.713015 |
| H | 2.355187 | 2.834839 | 0.813381 |
| C | 0.223036 | 2.623166 | 0.752511 |
| C | -0.853329 | 1.752051 | 0.668017 |
| H | -1.862989 | 2.136091 | 0.728307 |
| C | 3.191678 | 0.288973 | 0.711903 |
| H | 3.299634 | -0.431678 | 1.524632 |
| H | 3.843605 | 1.138090 | 0.914467 |
| N | 3.782412 | -0.446652 | -0.497626 |

| | | | |
|---|---|---|---|
| C | 5.274949 | -0.620303 | -0.237094 |
| H | 5.691033 | 0.381828 | -0.119972 |
| H | 5.330893 | -1.136236 | 0.720602 |
| C | 6.004687 | -1.404992 | -1.313342 |
| H | 7.027087 | -1.574436 | -0.966470 |
| H | 6.061202 | -0.872067 | -2.264067 |
| H | 5.539192 | -2.373816 | -1.486977 |
| C | 3.501408 | 0.267409 | -1.808553 |
| H | 2.416414 | 0.343246 | -1.857983 |
| H | 3.816360 | -0.427085 | -2.584382 |
| C | 4.156774 | 1.632549 | -1.968432 |
| H | 3.828262 | 2.048547 | -2.923950 |
| H | 5.246314 | 1.575759 | -1.997834 |
| H | 3.860459 | 2.339730 | -1.192014 |
| H | -0.190706 | -2.108096 | 1.518836 |
| H | 1.808002 | -1.630496 | -0.022748 |
| H | 3.755492 | -2.733312 | 0.947443 |
| O | 4.148682 | -2.842692 | 1.833115 |
| H | 3.880626 | -3.709977 | 2.149698 |
| H | 3.261443 | -2.565285 | -2.223846 |
| O | 3.458484 | -2.835418 | -3.140985 |
| H | 2.739796 | -3.415028 | -3.408242 |
| H | -1.417267 | -3.796625 | 2.972837 |
| O | -1.699186 | -4.622307 | 3.398357 |
| H | -1.736961 | -4.422528 | 4.337881 |

# Sets of coordinates obtained at the ωB97XD/6-311++G(d,p) level of theory

## Compound 2

### Model A

48

scf done: -6240.826831

| | | | |
|---|---|---|---|
| Br | 4.093683 | 2.276854 | -4.771849 |
| Br | 6.640192 | 7.296014 | 0.338113 |
| O | 9.069263 | 1.206017 | -1.532115 |
| O | 10.283171 | 3.396084 | -2.448922 |
| O | 12.324117 | 4.726674 | -3.121703 |
| C | 7.497425 | 2.828417 | -2.423434 |
| C | 7.968065 | 1.514423 | -2.254650 |
| C | 7.254356 | 0.448743 | -2.802033 |
| H | 7.634138 | -0.552579 | -2.639370 |
| C | 6.099410 | 0.656759 | -3.534869 |
| H | 5.558976 | -0.181469 | -3.956372 |
| C | 5.656108 | 1.957308 | -3.735067 |
| C | 6.342136 | 3.026232 | -3.188604 |
| H | 5.985287 | 4.035450 | -3.355855 |
| C | 8.111718 | 4.016591 | -1.771256 |
| C | 9.489691 | 4.282366 | -1.819755 |
| C | 10.017845 | 5.435590 | -1.216695 |
| C | 9.156144 | 6.330828 | -0.588366 |
| H | 9.554053 | 7.217615 | -0.108374 |
| C | 7.797347 | 6.073875 | -0.541562 |
| C | 7.280177 | 4.925455 | -1.116647 |
| H | 6.218799 | 4.719306 | -1.053094 |
| C | 11.499791 | 5.690752 | -1.133055 |
| H | 12.033442 | 4.839830 | -0.703746 |
| H | 11.679393 | 6.563251 | -0.504756 |
| N | 12.206634 | 5.937068 | -2.454111 |
| C | 13.591074 | 6.451513 | -2.133825 |
| H | 13.463235 | 7.370259 | -1.558203 |
| H | 14.030244 | 5.693470 | -1.485151 |
| C | 14.452271 | 6.678130 | -3.357825 |

| | | | |
|---|---|---|---|
| H | 15.458727 | 6.930730 | -3.017805 |
| H | 14.092923 | 7.499998 | -3.979832 |
| H | 14.512338 | 5.780544 | -3.971589 |
| C | 11.439510 | 6.877948 | -3.345584 |
| H | 10.466066 | 6.408002 | -3.480387 |
| H | 11.951002 | 6.850308 | -4.306182 |
| C | 11.300048 | 8.293962 | -2.818816 |
| H | 10.662316 | 8.846999 | -3.511053 |
| H | 12.257751 | 8.815574 | -2.769225 |
| H | 10.822207 | 8.333634 | -1.838404 |
| H | 9.749867 | 1.875568 | -1.704525 |
| H | 11.139450 | 3.847234 | -2.745147 |
| H | 13.445832 | 3.639125 | -2.110145 |
| O | 13.928502 | 3.378855 | -1.306563 |
| H | 14.090279 | 2.438206 | -1.373556 |
| H | 12.599639 | 4.860941 | -4.915014 |
| O | 12.780549 | 5.159358 | -5.824007 |
| H | 12.342082 | 4.533096 | -6.399405 |

**Model B**

51

scf done: -6317.277158

| | | | |
|---|---|---|---|
| Br | -5.320206 | -0.870223 | -1.238296 |
| Br | -0.094978 | 4.579656 | -0.015538 |
| O | -1.013985 | -1.491611 | 2.941602 |
| O | 0.882026 | -1.264931 | 1.056506 |
| O | 3.116070 | -1.853631 | 0.186834 |
| C | -1.788152 | -0.381295 | 0.927194 |
| C | -1.938531 | -1.309228 | 1.972137 |
| C | -3.111059 | -2.059145 | 2.066832 |
| H | -3.203409 | -2.754500 | 2.892189 |
| C | -4.125088 | -1.928770 | 1.134835 |
| H | -5.026451 | -2.522655 | 1.219476 |
| C | -3.960973 | -1.038775 | 0.080584 |
| C | -2.812577 | -0.274790 | -0.021682 |
| H | -2.693889 | 0.413614 | -0.850086 |

| | | | |
|---|---|---|---|
| C | -0.618972 | 0.528024 | 0.787662 |
| C | 0.699855 | 0.050813 | 0.815391 |
| C | 1.778318 | 0.912906 | 0.567366 |
| C | 1.523218 | 2.257280 | 0.310333 |
| H | 2.348142 | 2.935789 | 0.124683 |
| C | 0.224821 | 2.737424 | 0.312106 |
| C | -0.838957 | 1.883345 | 0.547012 |
| H | -1.853052 | 2.263840 | 0.546803 |
| C | 3.215860 | 0.462052 | 0.623283 |
| H | 3.485698 | 0.072303 | 1.607318 |
| H | 3.863675 | 1.309555 | 0.398854 |
| N | 3.591405 | -0.656140 | -0.331712 |
| C | 5.098845 | -0.732197 | -0.375035 |
| H | 5.458823 | 0.235117 | -0.730049 |
| H | 5.404033 | -0.859248 | 0.663968 |
| C | 5.613805 | -1.868664 | -1.232817 |
| H | 6.693361 | -1.941872 | -1.087243 |
| H | 5.429218 | -1.709421 | -2.296658 |
| H | 5.162262 | -2.814575 | -0.935781 |
| C | 2.989807 | -0.472944 | -1.702483 |
| H | 1.911102 | -0.460956 | -1.550764 |
| H | 3.216842 | -1.389655 | -2.243367 |
| C | 3.460555 | 0.762977 | -2.444525 |
| H | 2.907653 | 0.816353 | -3.384192 |
| H | 4.523111 | 0.722219 | -2.692758 |
| H | 3.256779 | 1.685409 | -1.897255 |
| H | -0.132509 | -1.452096 | 2.539768 |
| H | 1.826404 | -1.570902 | 0.748931 |
| H | 3.979950 | -2.163158 | 1.798724 |
| O | 4.511190 | -1.899337 | 2.569983 |
| H | 4.426435 | -2.598305 | 3.217814 |
| H | 2.701357 | -3.220275 | -1.131093 |
| O | 2.389401 | -3.670367 | -1.927589 |
| H | 1.464916 | -3.398423 | -1.981624 |
| H | -0.040909 | -2.106787 | -0.493453 |

| | | | |
|---|---|---|---|
| O | -0.146096 | -2.265515 | -1.440608 |
| H | -1.091438 | -2.339502 | -1.581023 |

**Model C**

51

scf done: -6317.270108

| | | | |
|---|---|---|---|
| Br | -5.371614 | -0.119916 | -1.786743 |
| Br | 0.066570 | 4.475800 | 0.929887 |
| O | -1.140313 | -2.129430 | 2.018470 |
| O | 0.817851 | -1.473272 | 0.392483 |
| O | 3.120703 | -1.792734 | -0.572003 |
| C | -1.839331 | -0.465889 | 0.401014 |
| C | -2.058197 | -1.640743 | 1.136811 |
| C | -3.263623 | -2.328472 | 1.023283 |
| H | -3.403030 | -3.226693 | 1.614571 |
| C | -4.258726 | -1.879663 | 0.171637 |
| H | -5.191283 | -2.423068 | 0.087281 |
| C | -4.034862 | -0.736097 | -0.582943 |
| C | -2.845313 | -0.039028 | -0.473114 |
| H | -2.683269 | 0.846828 | -1.075273 |
| C | -0.627403 | 0.385098 | 0.545083 |
| C | 0.674231 | -0.140434 | 0.516355 |
| C | 1.790308 | 0.704642 | 0.625665 |
| C | 1.594835 | 2.078714 | 0.735323 |
| H | 2.448350 | 2.739989 | 0.832096 |
| C | 0.313371 | 2.600997 | 0.758872 |
| C | -0.786971 | 1.764752 | 0.675696 |
| H | -1.786447 | 2.179589 | 0.721002 |
| C | 3.193673 | 0.168828 | 0.737109 |
| H | 3.280522 | -0.571363 | 1.535779 |
| H | 3.876257 | 0.990925 | 0.952474 |
| N | 3.729864 | -0.548927 | -0.488498 |
| C | 5.213136 | -0.749121 | -0.278590 |
| H | 5.652427 | 0.242266 | -0.152103 |
| H | 5.293097 | -1.287972 | 0.665700 |

| | | | |
|---|---|---|---|
| C | 5.886548 | -1.516109 | -1.396381 |
| H | 6.918467 | -1.709403 | -1.096083 |
| H | 5.909924 | -0.959351 | -2.334976 |
| H | 5.395522 | -2.471809 | -1.573859 |
| C | 3.427342 | 0.194795 | -1.762850 |
| H | 2.342221 | 0.288368 | -1.783773 |
| H | 3.711942 | -0.479748 | -2.568508 |
| C | 4.100123 | 1.548461 | -1.892359 |
| H | 3.739256 | 2.014852 | -2.811025 |
| H | 5.185857 | 1.467659 | -1.971798 |
| H | 3.852788 | 2.221949 | -1.069905 |
| H | -0.258825 | -2.073560 | 1.607293 |
| H | 1.735211 | -1.691850 | 0.014793 |
| H | 3.654636 | -2.779328 | 0.923267 |
| O | 4.029129 | -2.877330 | 1.815246 |
| H | 3.734094 | -3.724590 | 2.148177 |
| H | 3.167993 | -2.563920 | -2.223943 |
| O | 3.350646 | -2.793493 | -3.151978 |
| H | 2.600306 | -3.306725 | -3.450537 |
| H | -1.619599 | -3.665321 | 3.025938 |
| O | -2.019796 | -4.451128 | 3.422815 |
| H | -2.129540 | -4.236479 | 4.348925 |

**References:**

[1] Rospenk, M.; Koll, A.; Głowiak, T.; Sobczyk, L. *J. Mol. Struct.* **1989**, *195*, 33-41.

[2] Wojciechowski, G.; Ratajczak-Sitarz, M.; Katrusiak, A.; Brzeziński, B. *J. Mol. Struct.* **2002**, *612*, 59-64.
